# Identification of exosomal miRNAs as diagnostic biomarkers for cholangiocarcinoma and gallbladder carcinoma

**DOI:** 10.1038/s41392-020-0162-6

**Published:** 2020-06-12

**Authors:** Xin-ying Xue, Yu-xia Liu, Chen Wang, Xin-jin Gu, Zhi-qiang Xue, Xue-lei Zang, Xi-dong Ma, Hui Deng, Rong Liu, Lei Pan, San-hong Liu

**Affiliations:** 1 Department of Respiratory and Critical Care Medicine, Beijing Shijitan Hospital, Capital Medical University, Beijing, China; 2 Department of Respiratory and Critical Care Medicine, General Hospital of PLA, Beijing, China; 3 Department of Respiratory and Critical Care Medicine, Hospital of Weifang Medical University, Shandong, China; 4 Department of Scientific Research, Peking Union Medical College Hospital, Beijing, China; 5 Shanghai Institute for Advanced Immunochemical Studies, ShanghaiTech University, Shanghai, China; 6 Department of Hepatobiliary and Pancreatic Oncology Surgery, General Hospital of PLA, Beijing, China; 7 Department of Thoracic Surgery, General Hospital of PLA, Beijing, China; 8 Department of Laboratory, General Hospital of PLA, Beijing, China

**Keywords:** Predictive markers, Tumour biomarkers, Tumour biomarkers

**Dear Editor**,

Extracellular vesicles, especially exosomes, have emerged as promising diagnostic sources for cancers due to their easy and quick accessibility.^1,2^ Additionally, it has been demonstrated that circulating miRNAs serve as promising biomarkers for the diagnosis of multiple diseases.^3,4^ Cholangiocarcinoma (CCA) and gallbladder carcinoma (GBC) are two malignant biliary tract cancers. It has been estimated that patients with unresectable GBC have a 5-year survival of only less than 5%, while CCA patients suffer from an overall 5-year survival of ~10%.^5^ Hence, circulating diagnostic biomarkers would benefit the diagnosis and treatment of GBC and CCA patients. We sequenced exosomal small RNA from five CCA patients and four GBC patients before and after surgery and enrolled 40 healthy individuals, 45 more CCA patients and 24 more GBC patients to validate the sequencing results to identify diagnostic biomarkers for GBC and CCA.

To identify small RNAs that are specifically present in exosomes from the blood of CCA and GBC patients, 50 healthy individuals, five CCA patients and four GBC patients were enrolled (Supplementary Table S1). Exosomes were extracted and examined by electron microscopy (Fig. 1a), and exosomal RNAs were sequenced as described previously.^6^ We first analyzed the small RNA length distribution and nucleotide preference of normal individuals and CCA and GBC patients before and after surgery. It is very interesting that for 15–23 nt small RNAs, 21 nt miRNAs were the most abundant in normal individuals. However, the most abundant miRNAs in CCA and GBC patients were 22 nt miRNAs. Moreover, the abundance of 15–20 nt small RNAs was lower in normal individuals but was increased in CCA and GBC patients and was decreased after surgery. In addition, the abundance of 30 nt small RNAs was the highest in normal individuals, while it was significantly reduced in CCA and GBC patients and was partially recovered after surgery (Supplementary Fig. S1). We also found no significant difference in nucleotide preference among normal individuals, CCA patients and GBC patients (Fig. 1b and Supplementary Fig. S2). We then annotated the exosomal RNAs and found that a large portion of protein coding genes existed in the apparently healthy individuals, but these protein coding genes were missing in both CCA and GBC patients regardless of surgery (Supplementary Fig. S3). Importantly, the portion of miRNAs was decreased as well in the exosomes of CCA and GBC patients (Supplementary Fig. S3), suggesting that the difference in expressed miRNA species and levels may serve as diagnostic markers for CCA and GBC. We then included the data of 50 normal controls by reanalyzing data from a published work.^6^ MiRNA expression density analysis showed that the density peaks of CCA- and GBC-derived exosomal miRNAs were shifted compared to those of the 50 normal controls (Supplementary Fig. S4). The expression correlation analysis showed that CCA patients and GBC patients were clustered together. These results suggest that patient-derived exosomal miRNAs share a common signature. The principal component analysis (PCA) of normal controls, CCA patients, and GBC patients revealed that the miRNA expression profiles in the exosomes of CCA and GBC patients were similar in comparison to those in the exosomes of lung cancer patients (Supplementary Fig. S6). Moreover, we analyzed differentially expressed miRNAs (Fig. 1d) and clustered the differentially expressed miRNAs with *p*-values less than 0.05 and over a two-fold change. Our results showed that samples from CCA or GBC patients were clustered together when normal controls were clustered together (Supplementary Fig. S7), suggesting that patient-derived exosomal miRNAs shared a common signature. Interestingly, we found that over 47% of differentially expressed exosomal miRNAs derived from CCA and GBC patients were the same (Fig. 1e). We next selected 10 upregulated miRNAs from the exosomes of CCA and GBC patients (Supplementary Fig. S8) and validated the upregulation in 40 more healthy individuals, 45 more CCA patients, and 24 more GBC patients. Our data showed that miR-96-5p, miR-151a-5p, miR-191-5p, and miR-4732-3p were significantly upregulated in the exosomes of CCA patients in comparison to the exosomes of normal controls (Fig. 1f). Receiver operating characteristic (ROC) analysis for these four miRNAs showed that the corresponding AUCs were 0.733, 0.7639, 0.5417, and 0.6544, respectively (Fig. 1g). However, the validation results of exosomal miRNAs in GBC patients did not agree with the sequencing results (Supplementary Fig. S10a, b). Importantly, we found that CCA patients in stage II had the highest exosomal levels of miR-96-5p, miR-151a-5p, miR-191-5p, and miR-4732-3p (Fig. 1h), suggesting that these markers are promising for early stage diagnosis. In addition, we found significantly decreased exosomal miR-151a-5p, miR-191-5p, and miR-4732-3p levels in CCA patients who underwent surgery (Fig. 1i). In contrast, stage III GBC patients had the highest exosomal levels of miR-182-5p, miR-191-5p, and miR-192-5p (Supplementary Fig. S10c). However, the levels of these miRNAs remained high after the patients underwent surgery (Supplementary Fig. S10d). Interestingly, we noticed that miR-9-3p, miR-129-5p, miR-218-5p, miR-433-3p, NC_000001.11_1920, and NC_000010.11_20947 were significantly downregulated in the exosomes of both CCA patients and GBC patients (Supplementary Fig. S9).

**Fig. 1:**
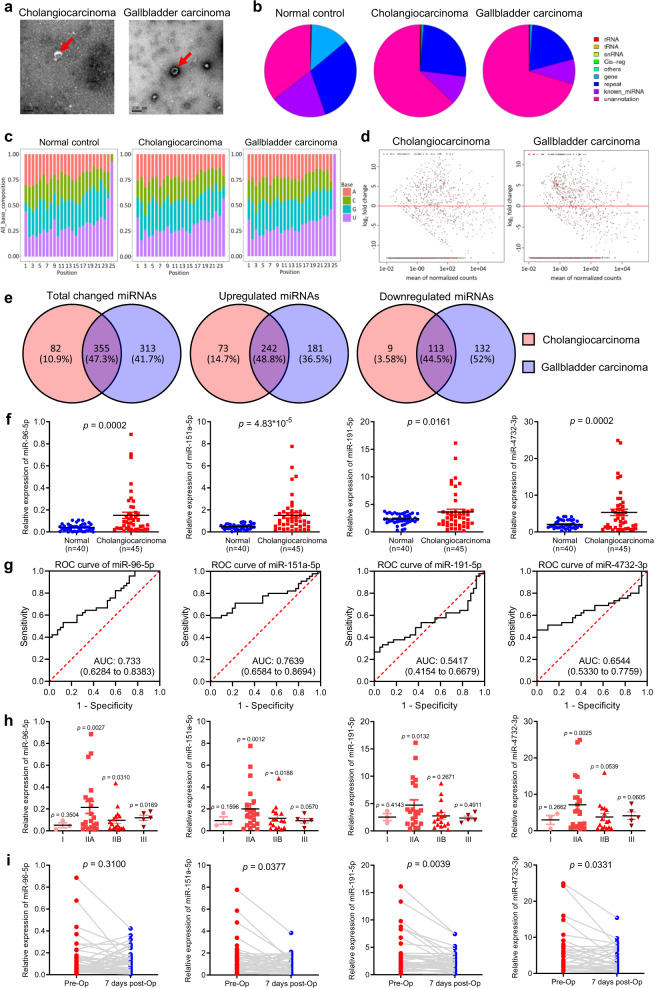
**a** Transmission electron micrograph of exosomes from CCA and GBC patients. **b** Annotations of the exosomal small RNAs of normal individuals, CCA patients, and GBC patients. **c** Nucleotide preference of the exosomal small RNAs of normal individuals, CCA patients, and GBC patients. **d** Volcano map showing differentially expressed miRNAs in CCA- and GBC-derived exosomes. **e** Venn diagram showing overlapping differentially expressed miRNAs in CCA- and GBC-derived exosomes. **f** Expression levels of miR-96-5p, miR-151a-5p, miR-191-5p, and miR-4732-3p in plasma from 40 healthy individuals and 45 CCA patients. **g** ROC curve analysis for CCA diagnosis. Area under the curve (AUC) estimation for miR-96-5p, miR-151a-5p, miR-191-5p, and miR-4732-3p in CCA patients and healthy individuals. **h** Expression levels of miR-96-5p, miR-151a-5p, miR-191-5p, and miR-4732-3p in plasma from 45 CCA patients according to their tumor stage. **i** Changes in the plasma levels of miR-96-5p, miR-151a-5p, miR-191-5p, and miR-4732-3p in 45 CCA patients before (pre-Op) and 7 days after (7 days post-Op) surgical removal of the tumor

To speculate the role of the differentially expressed exosomal RNAs in CCA patients, we analyzed the target genes of all the altered miRNAs and found that most of the target genes were enriched in cancer-related pathways, including acute myeloid leukemia, basal cell carcinoma, proteoglycans in cancer, and pathways in cancer (Supplementary Fig. S11a). Additionally, we analyzed the enrichment of the target genes of all the altered miRNAs in KEGG pathways and Gene Ontology (GO) terms in GBC patients and obtained similar results with CCA patients (Fig. S11a-d). Importantly, the target genes of miR-96-5p, miR-151a-5p, miR-191-5p, and miR-4732-3p were all enriched in the MAPK-signaling pathway and pathways in cancer (Supplementary Figs. S12 and S14a). Then, we analyzed the target genes of all the altered miRNAs and miR-96-5p, miR-151a-5p, miR-191-5p, and miR-4732-3p by GO (biological process) and found that neurogenesis was the shared enriched process for the targets of miR-96-5p, miR-151a-5p, miR-191-5p, and miR-4732-3p (Supplementary Figs. S13 and S14b).

In summary, we identified the differentially expressed exosomal miRNAs in CCA and GBC patients with high-throughput small RNA sequencing. After a larger-scale validation, we found that miR-96-5p, miR-151a-5p, miR-191-5p, and miR-4732-3p were significantly increased in the exosomes of CCA patients, while miR-151a-5p was slightly increased in the exosomes of GBC patients. In addition, it is interesting to note that there are significant differences in the small RNA length distribution among normal individuals, CCA patients, and GBC patients. Our data suggest a set of novel biomarkers for the diagnosis of CCA and GBC.

## Supplementary information

Supplemental

data S1

Data S2
