# Supplementary material for: Identification of exosomal miRNAs as diagnostic biomarkers for cholangiocarcinoma and gallbladder carcinoma
Source: Signal Transduct Target Ther. 2020 Jun 12;5:77. doi: 10.1038/s41392-020-0162-6 (PMC7289871; doi:10.1038/s41392-020-0162-6)
Supplement: Supplementary file 1 — Supplemental [file 41392_2020_162_MOESM1_ESM.docx]

Supplementary Materials for

**Identification of exosomal miRNAs as diagnostic biomarkers for cholangiocarcinoma and gallbladder carcinoma**

Xin-ying Xue^1,2,3^, Yu-xia Liu^4^, Chen Wang^5^, Xin-jin Gu^6^, Zhi-qiang Xue^7^, Xue-lei Zang^8^, Xi-dong Ma^3^, Hui Deng^1^, Rong Liu^6^, Lei Pan^1^, San-hong Liu^5^

Correspondence to: [liush@shanghaitech.edu.cn](mailto:liush@shanghaitech.edu.cn)

[leipan61@aliyun.com](mailto:leipan61@aliyun.com)

[liurong301@126.com](mailto:liurong301@126.com)

**This PDF file includes:**

Materials and Methods

Figures. S1 to S14

Tables S1

**Other Supplementary Materials for this manuscript include the following:**

Data S1 to S2

Materials and Methods

**Patient information**

Patient information were listed in Table 1. All patients signed consents for research study. All of the procedures were approved by the ethics committee of the Chinese PLA General Hospital and Beijing Shijitan Hospital of Capital Medical University in Beijing, and all experiments were conducted in accordance with the principles of the Declaration of Helsinki.

**Exosome separation and RNA isolation**

Peripheral blood sampling were performed on the day pathological examination confirmed CCA or GBC. We selected healthy controls with matched sex and age. Exosome separation and RNA isolation were performed according to our previous study ^1^.

**RNA Deep Sequencing**

For RNA sequencing, small RNAs were sent for library construction and were sequenced with an Illumina HiSeq3000 platform by OE biotech Co., Ltd. (Shanghai, China). HTSeq software was used for the statistics of the original miRNA deep sequencing data.

**Statistical analysis**

Statistical analysis were performed following the methods previously reported ^2^.

**Determination of plasma miRNA with quantitative PCR**

Quantitative PCR was performed according to our previous methods ^1^.


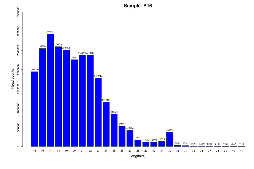

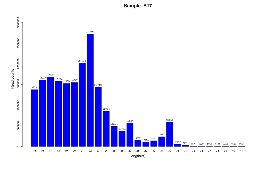

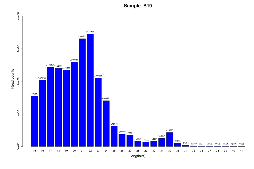

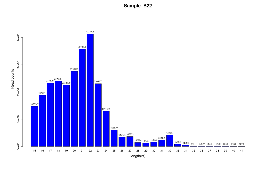

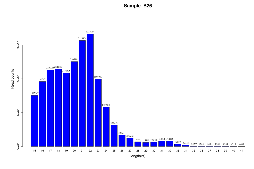

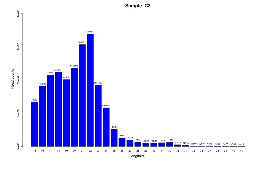

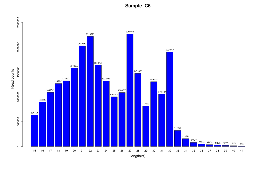

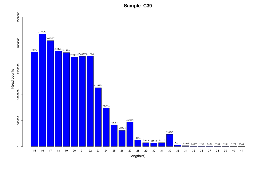

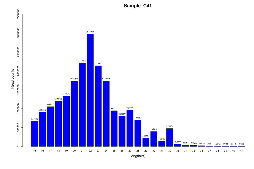

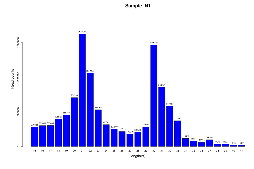

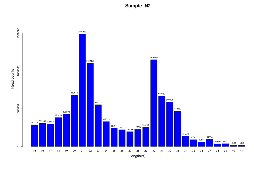

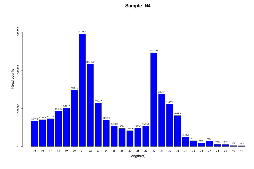

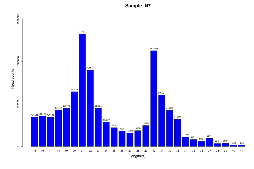

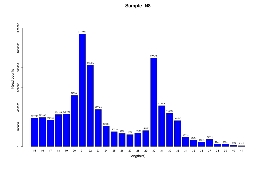

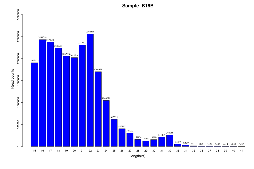

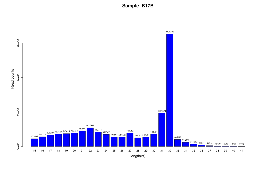

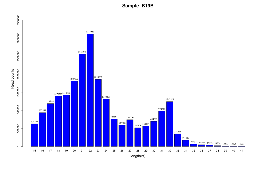

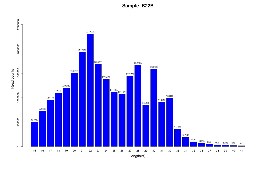

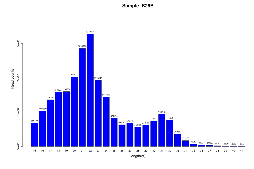

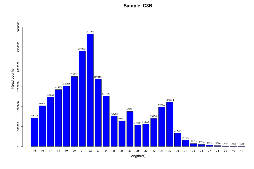

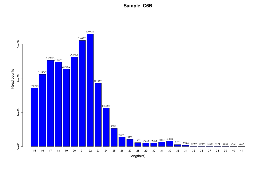

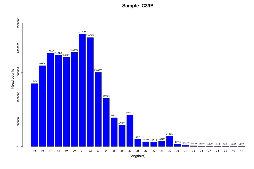

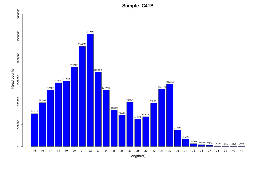


CCA

before surgery

CCA

after surgery

Normal

GBC

before surgery

GBC

after surgery

Figure. S1. Exosomal small RNA length distribution of normal individuals, CCA and GBC patients before and after surgeries


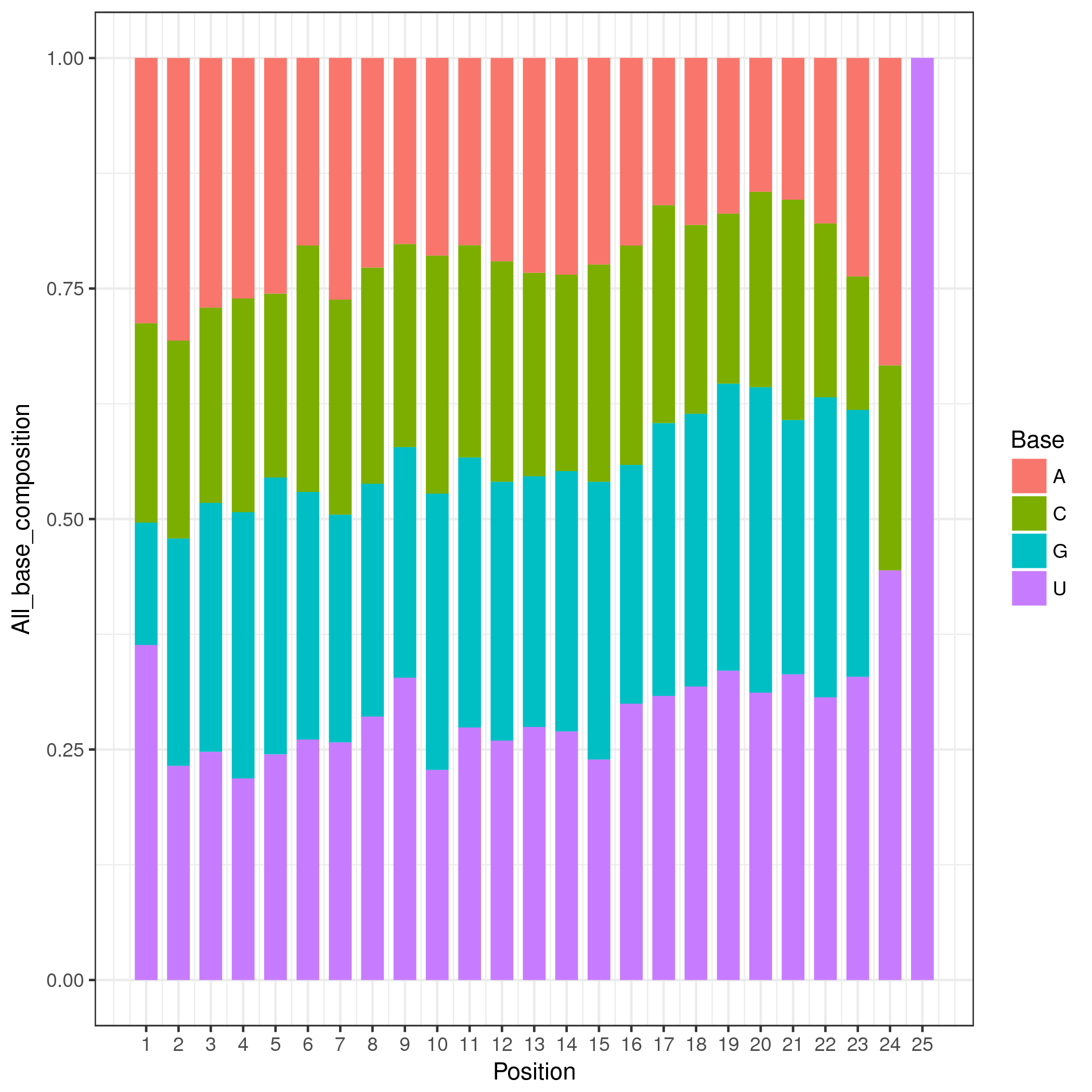

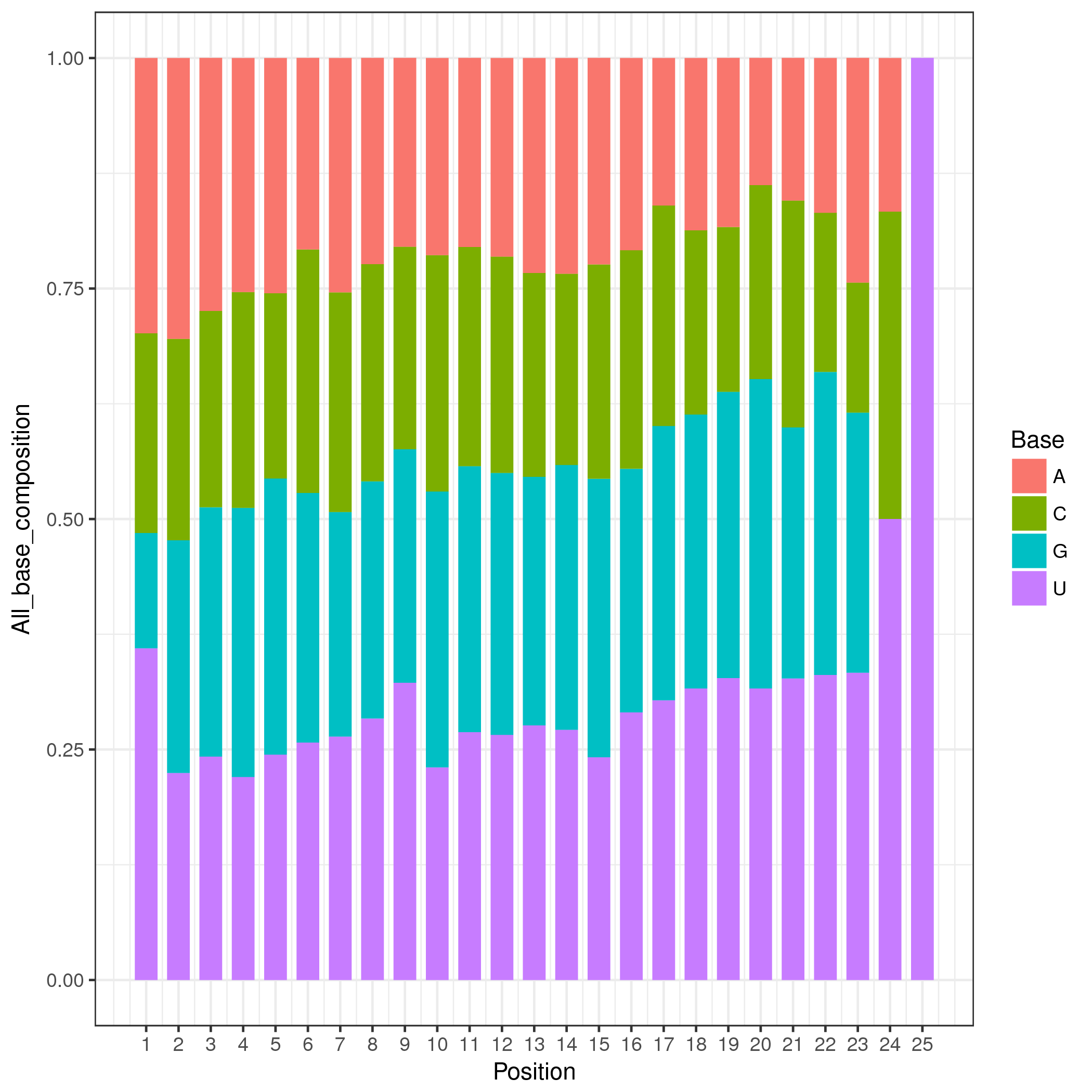

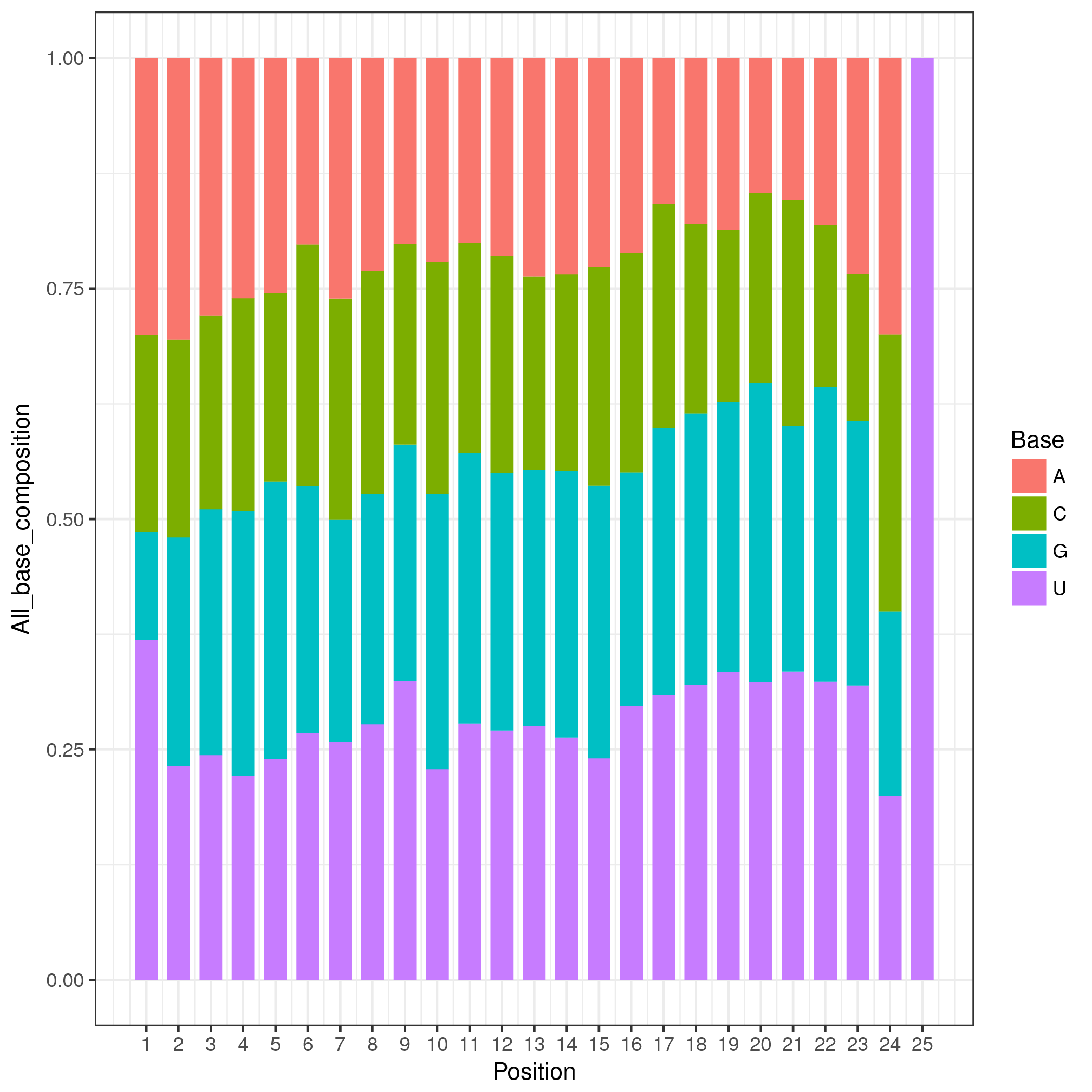

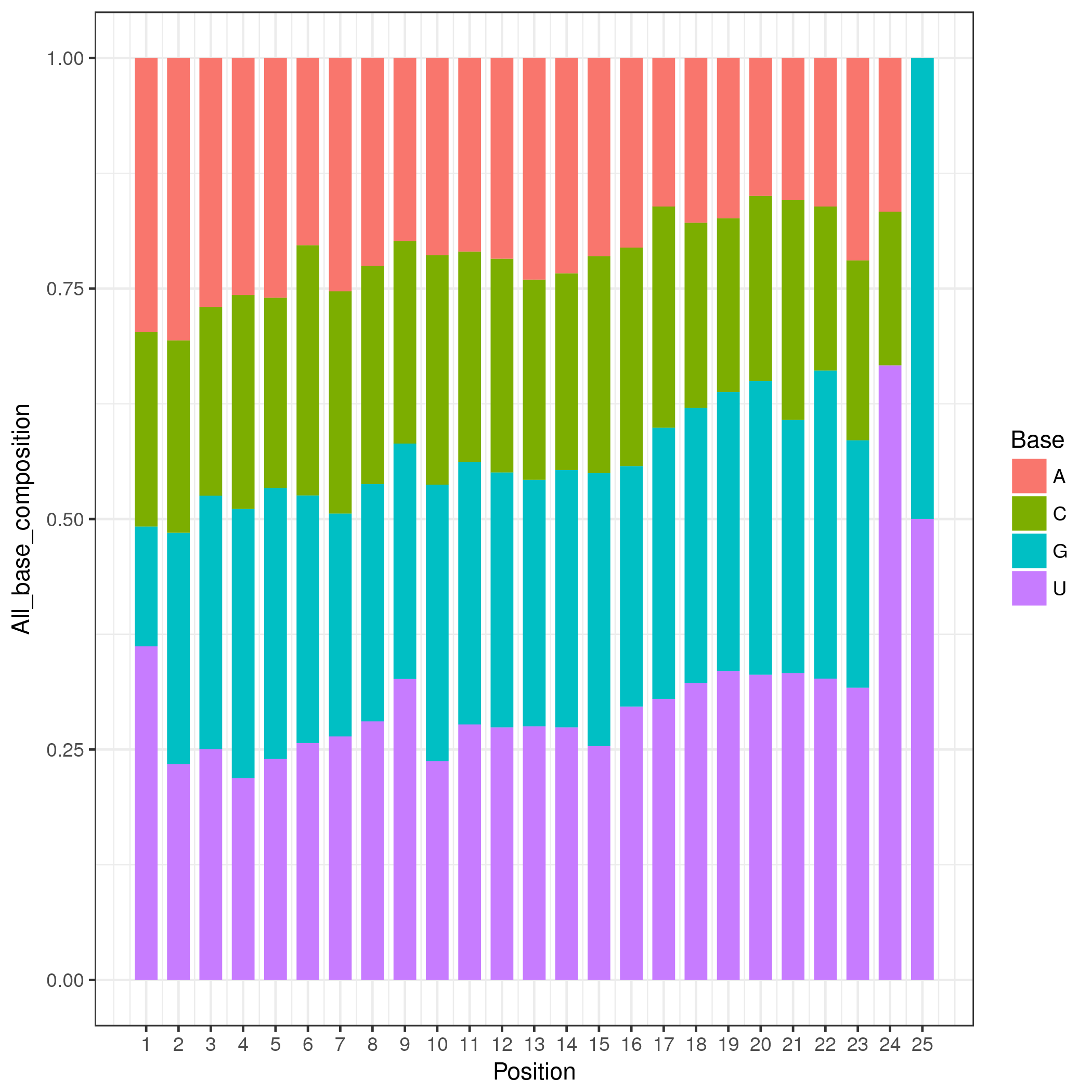

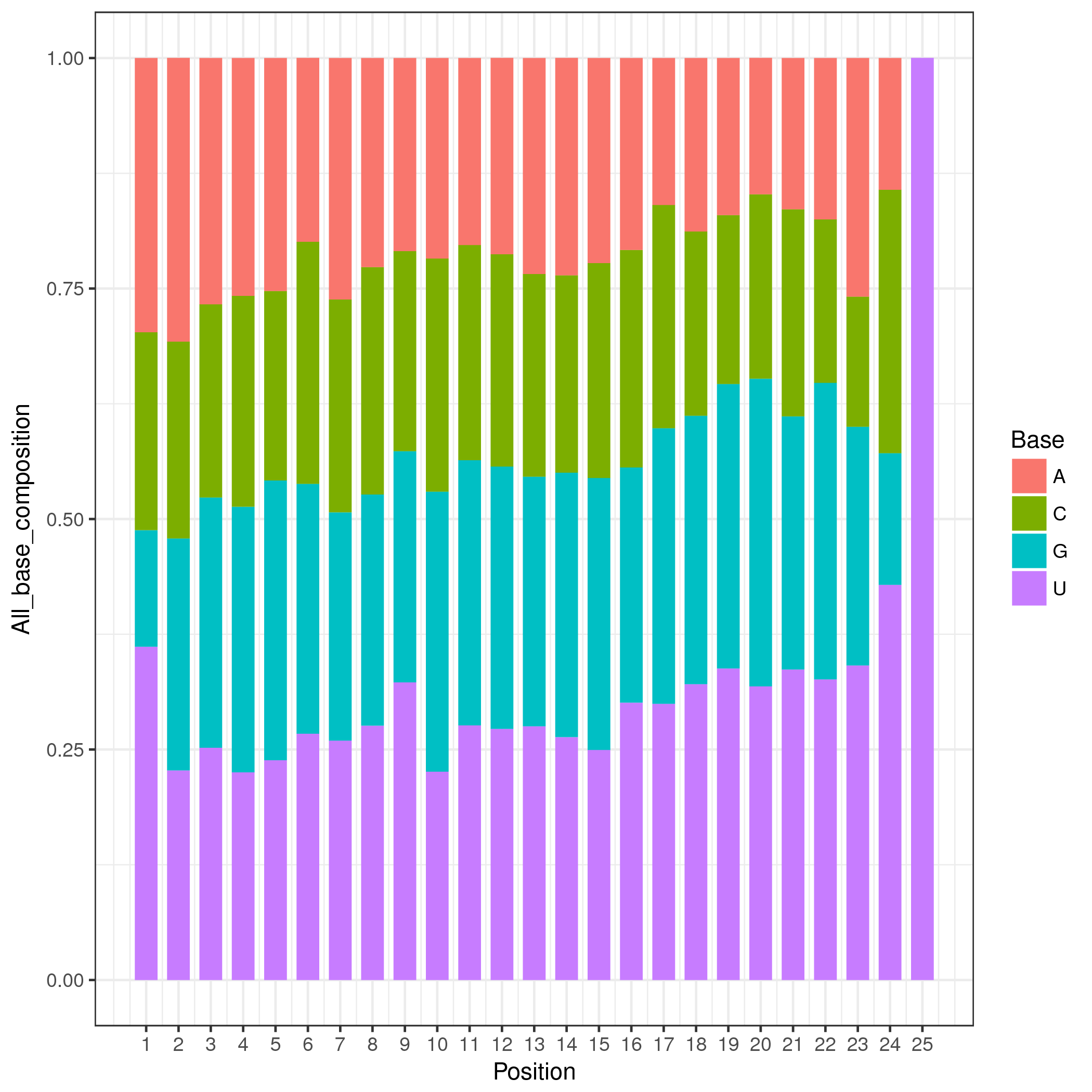

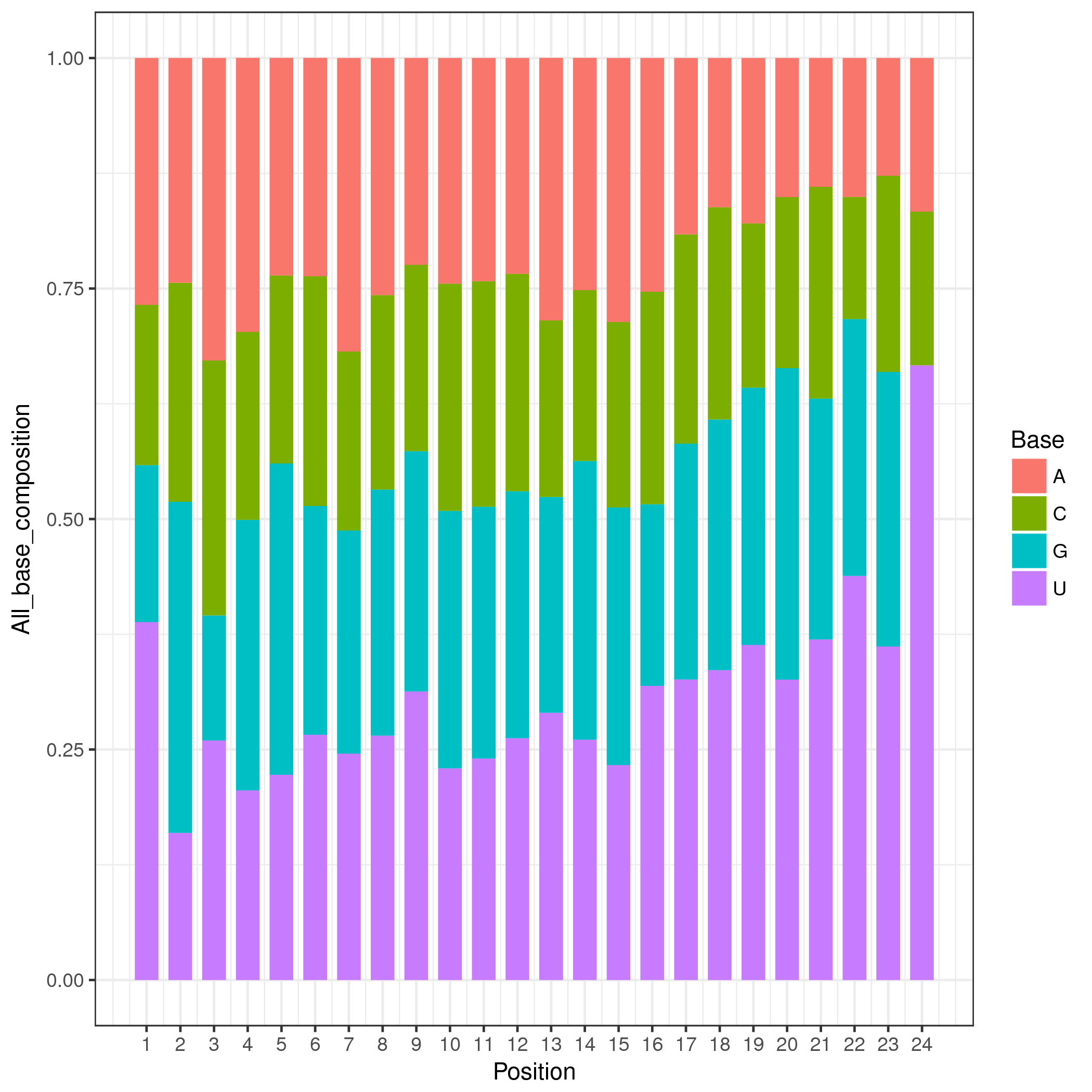

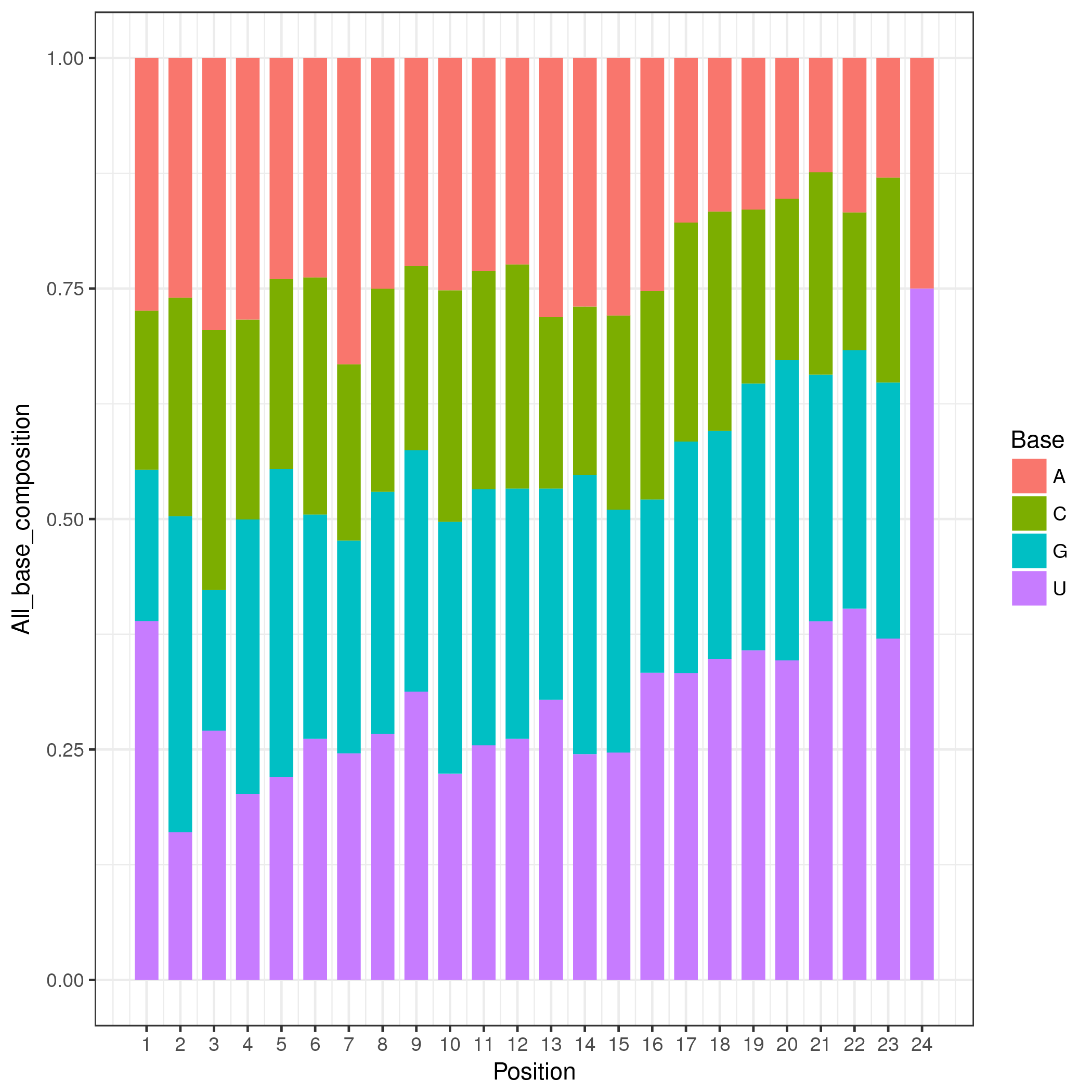

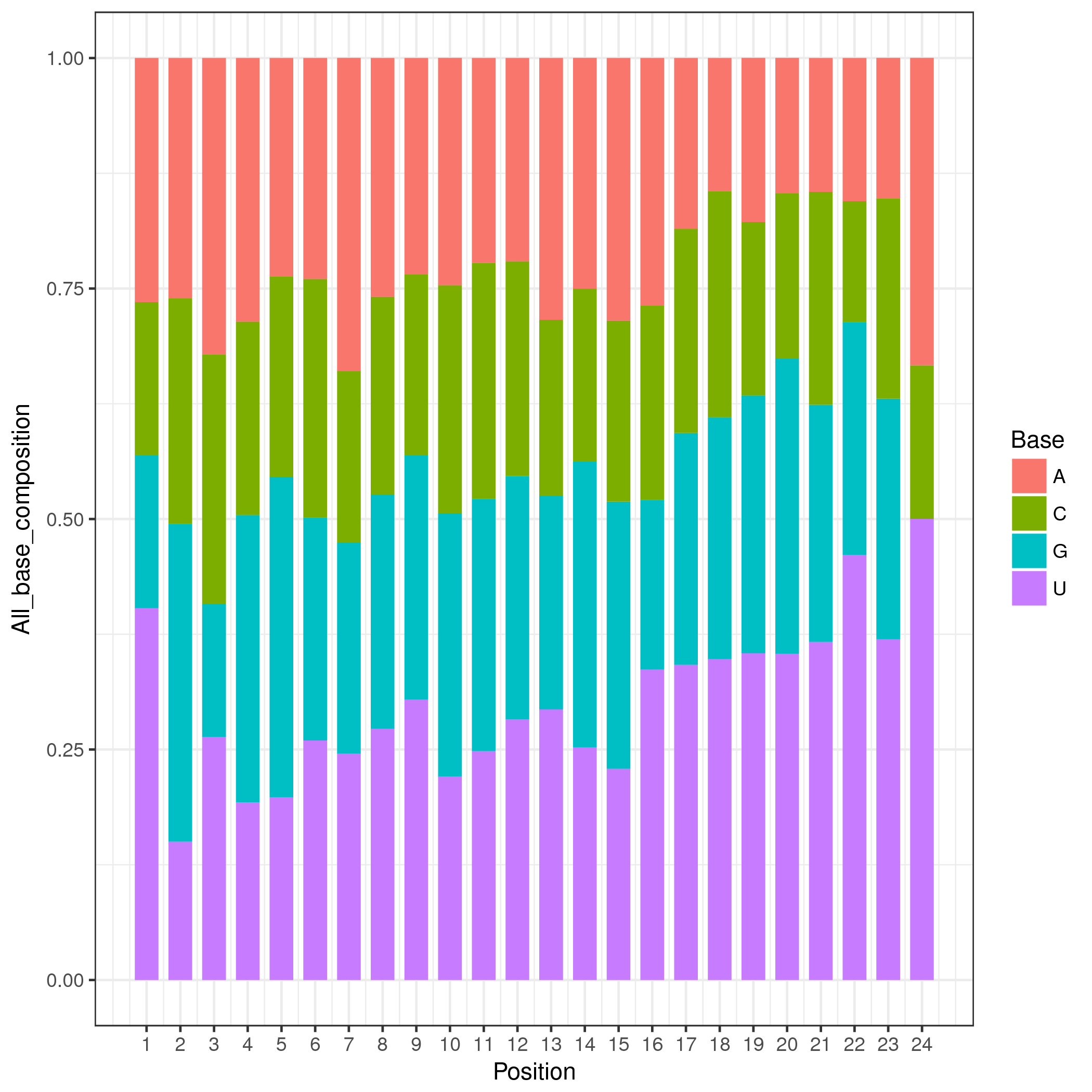

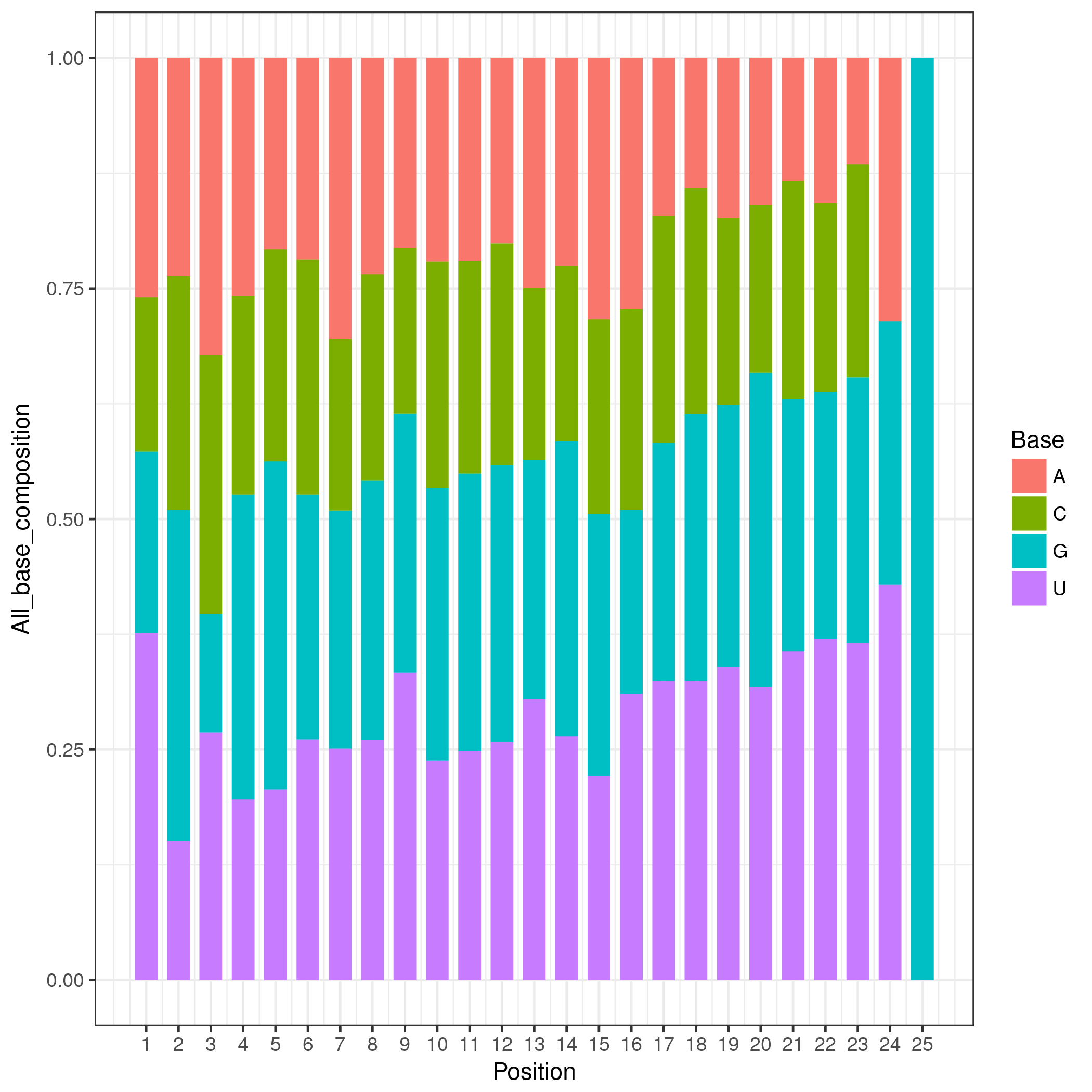

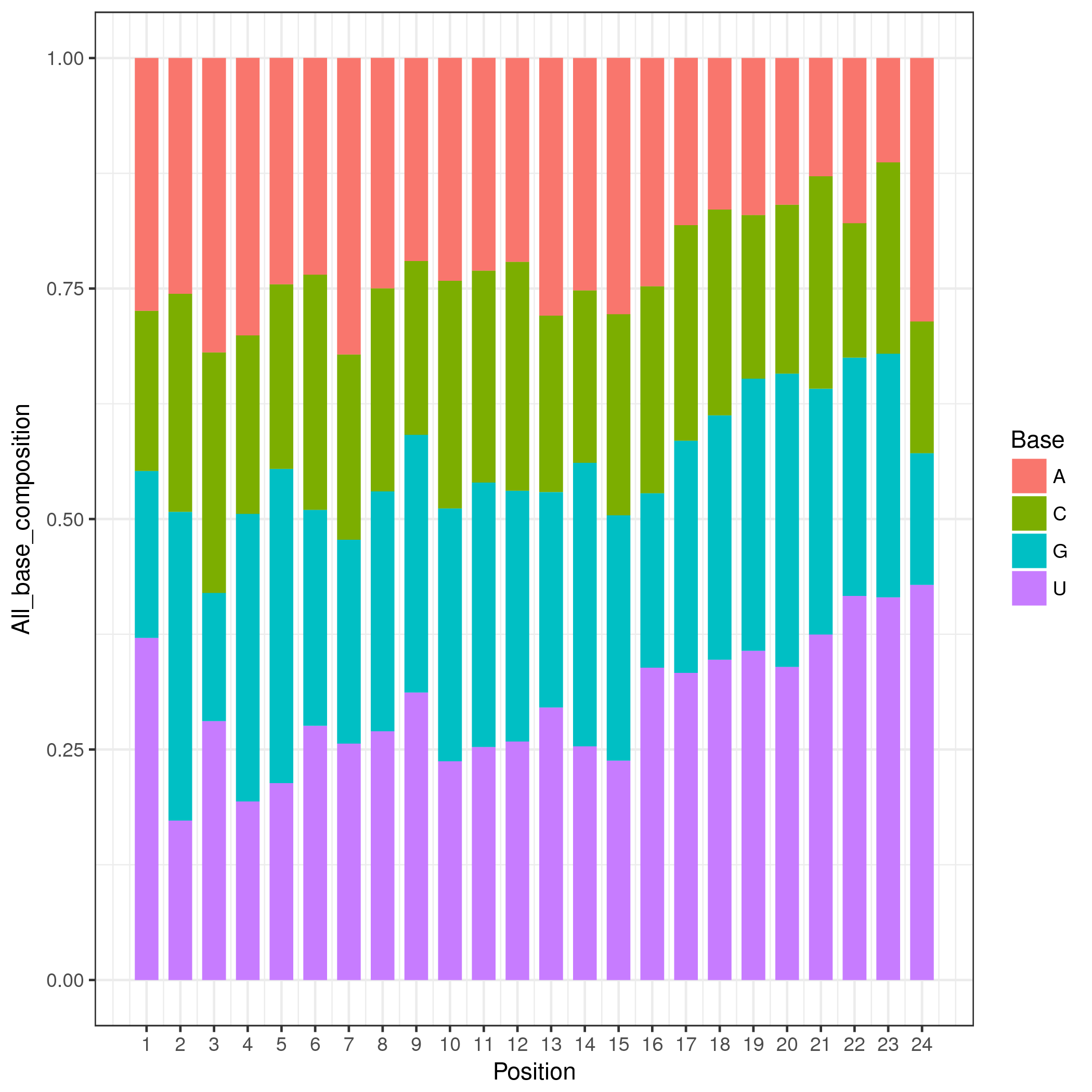

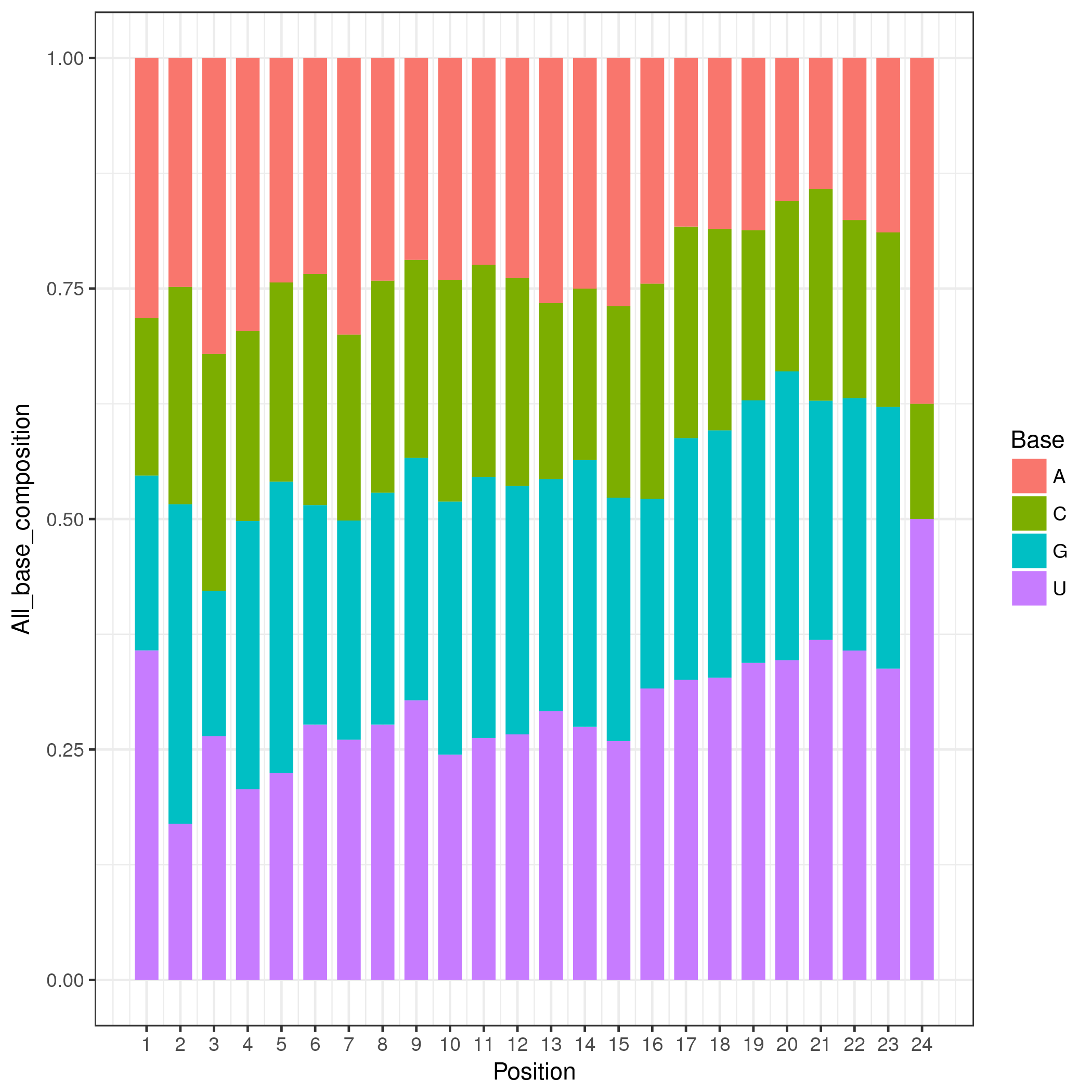

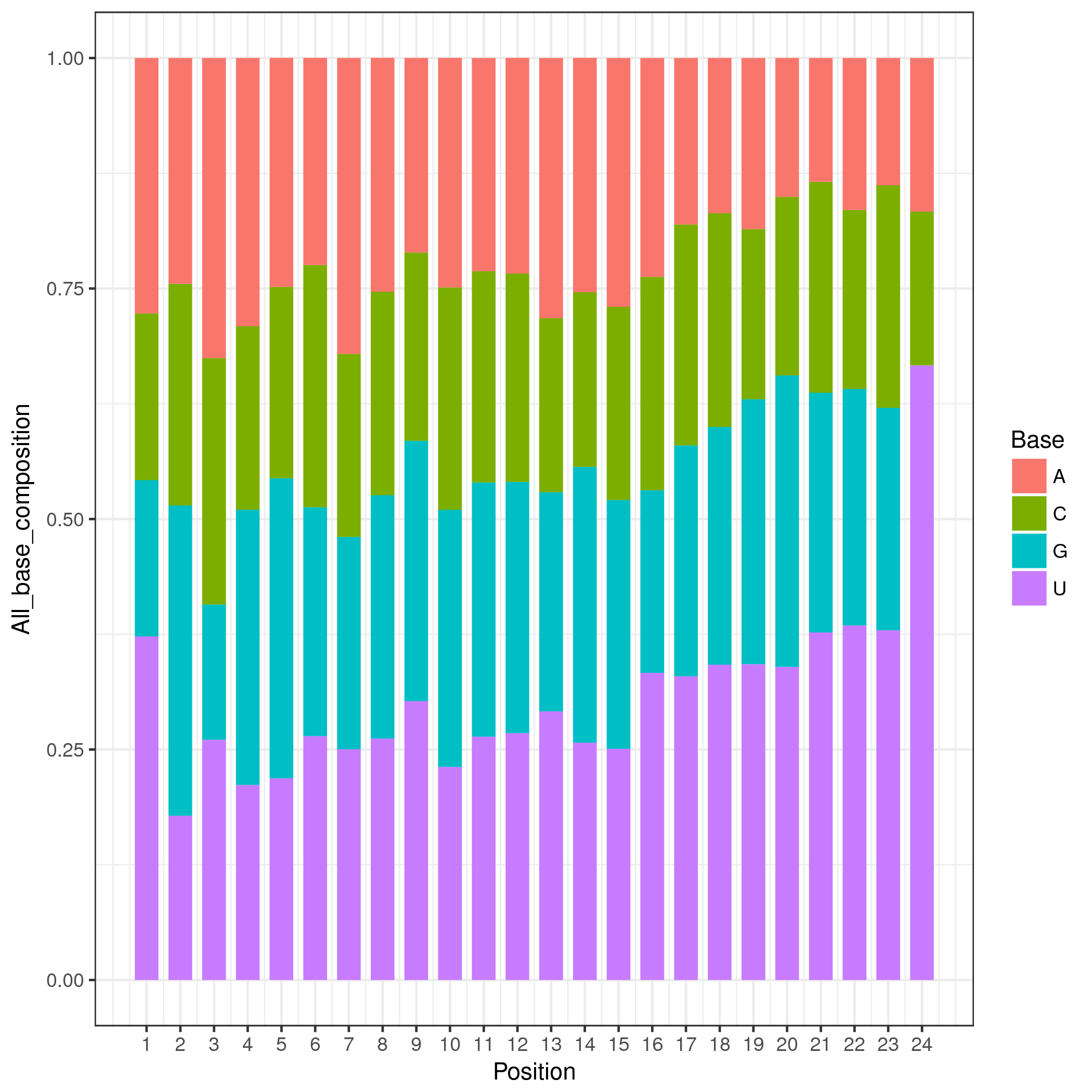

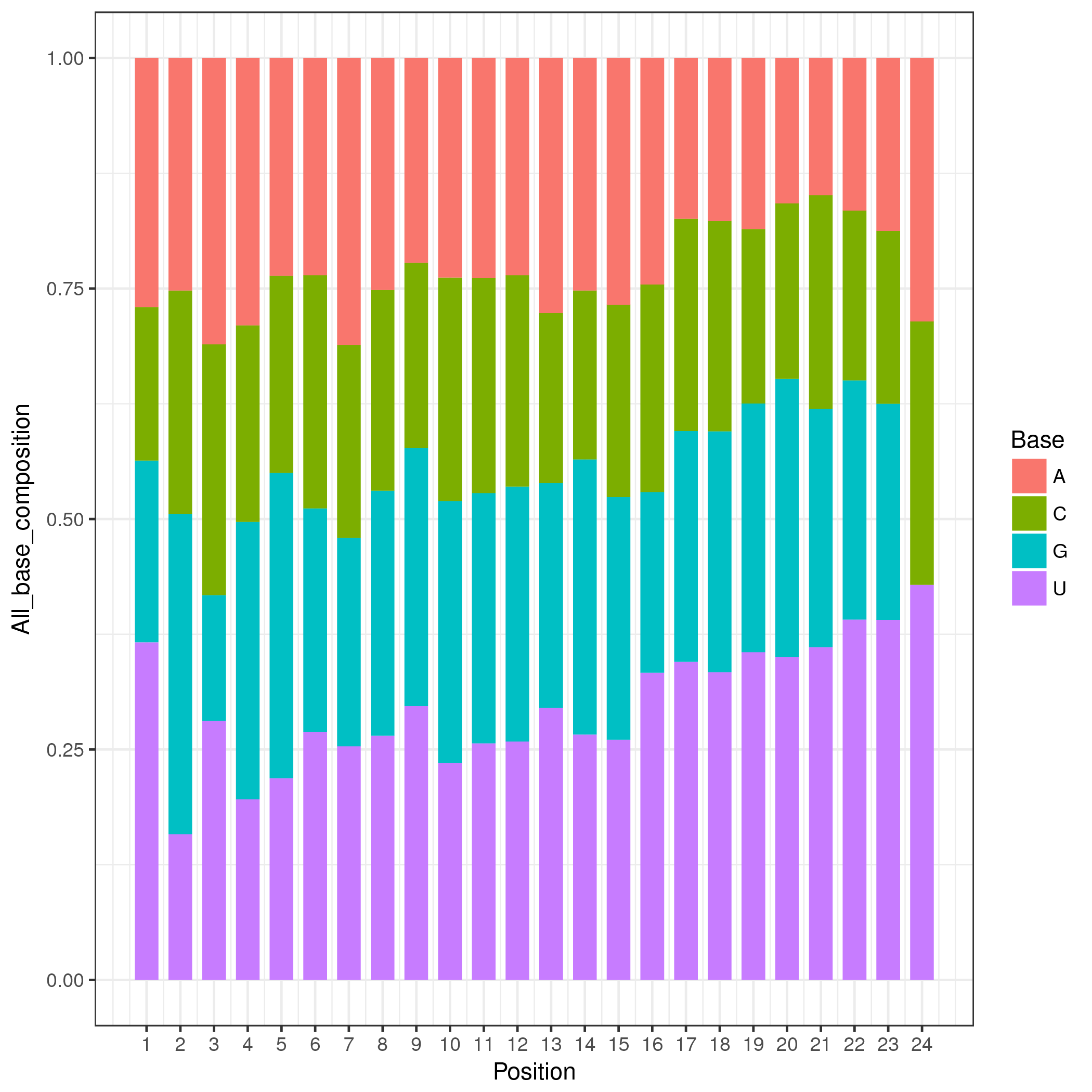

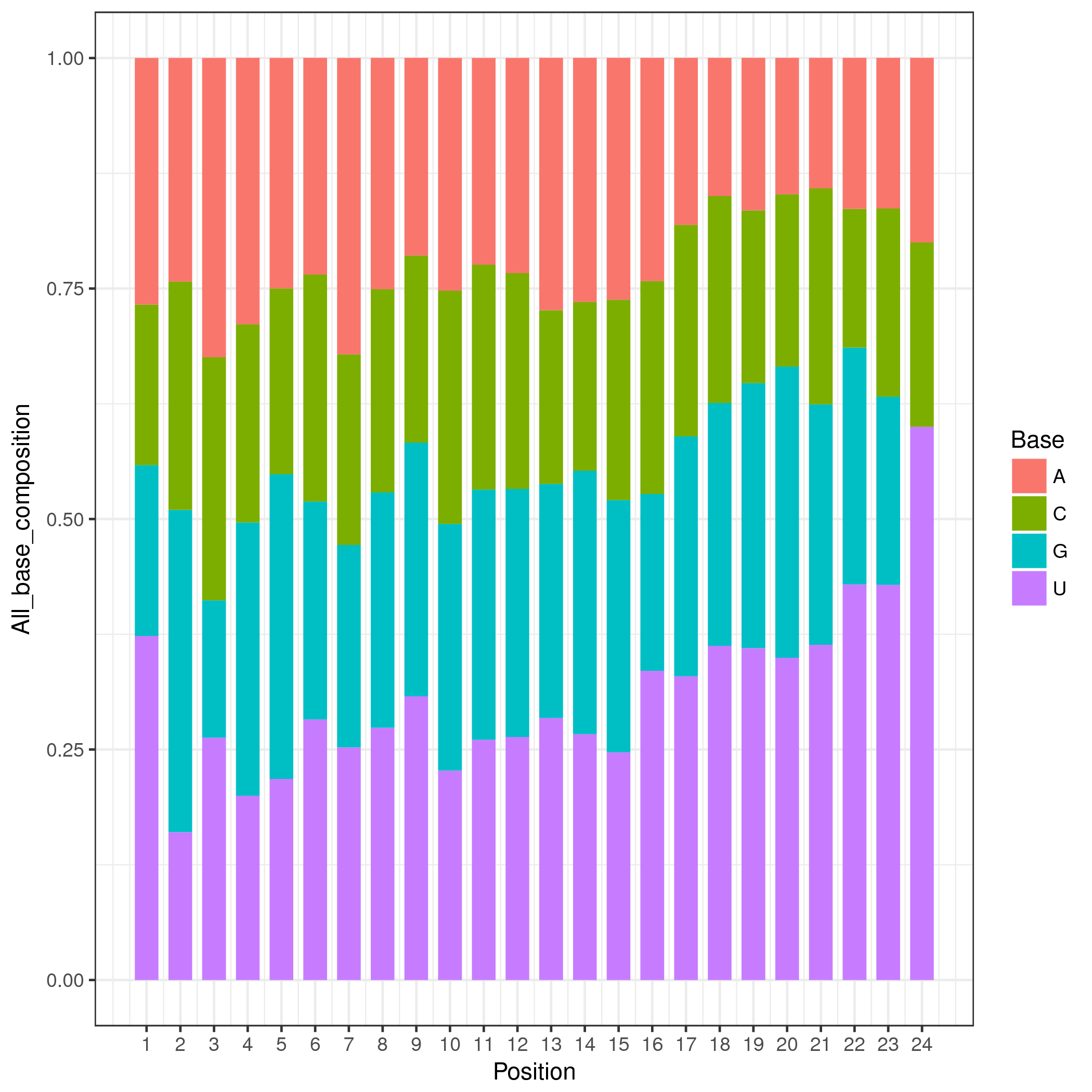

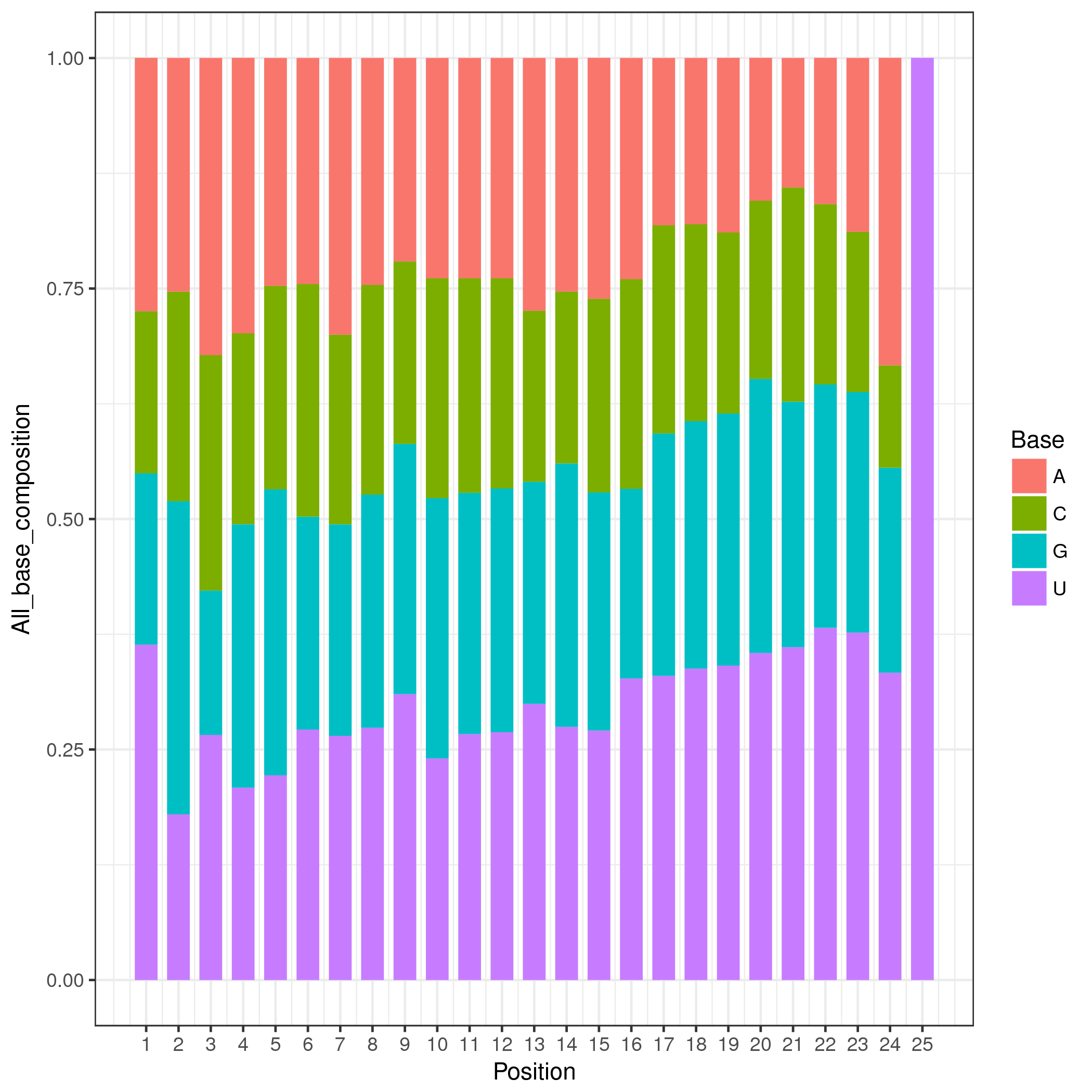

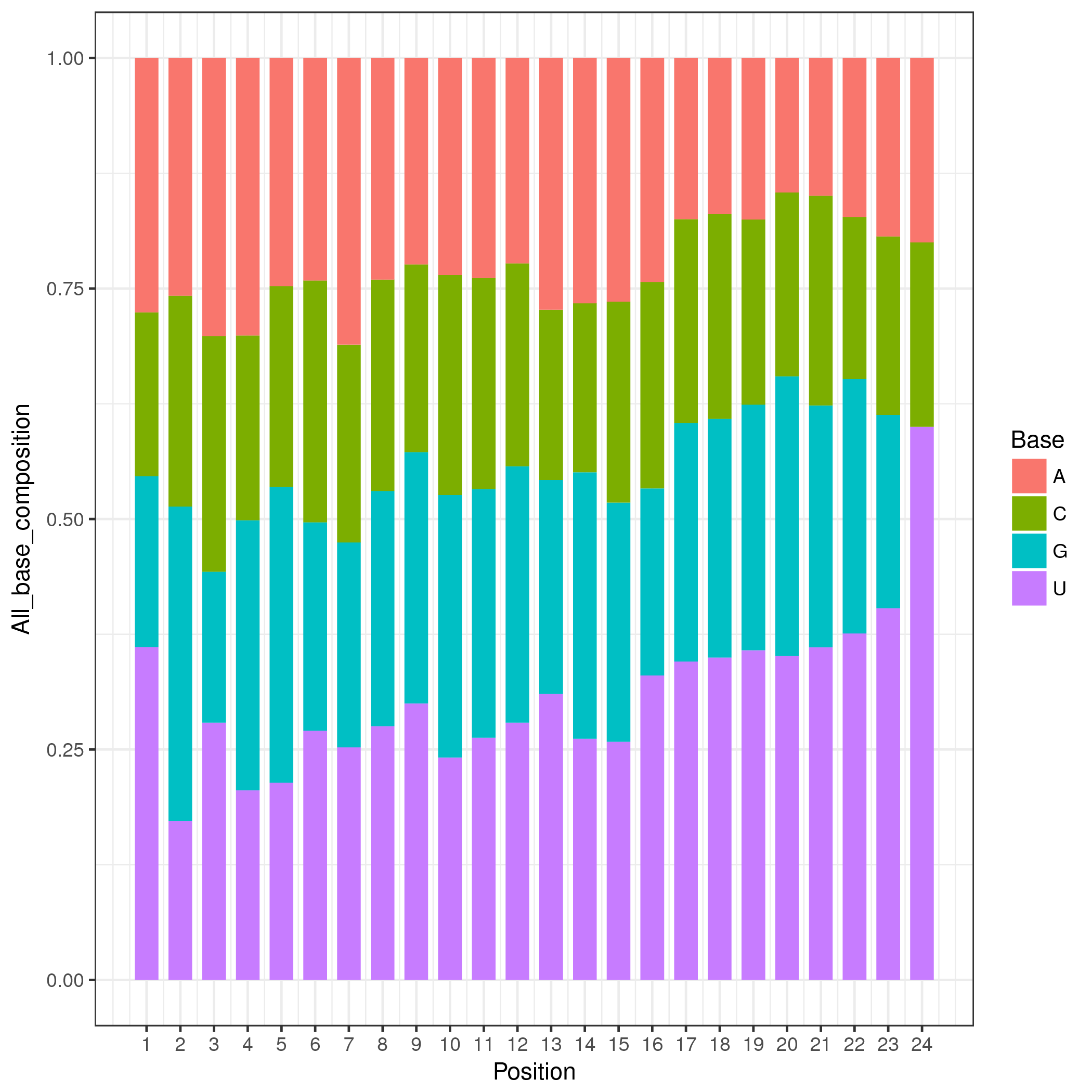

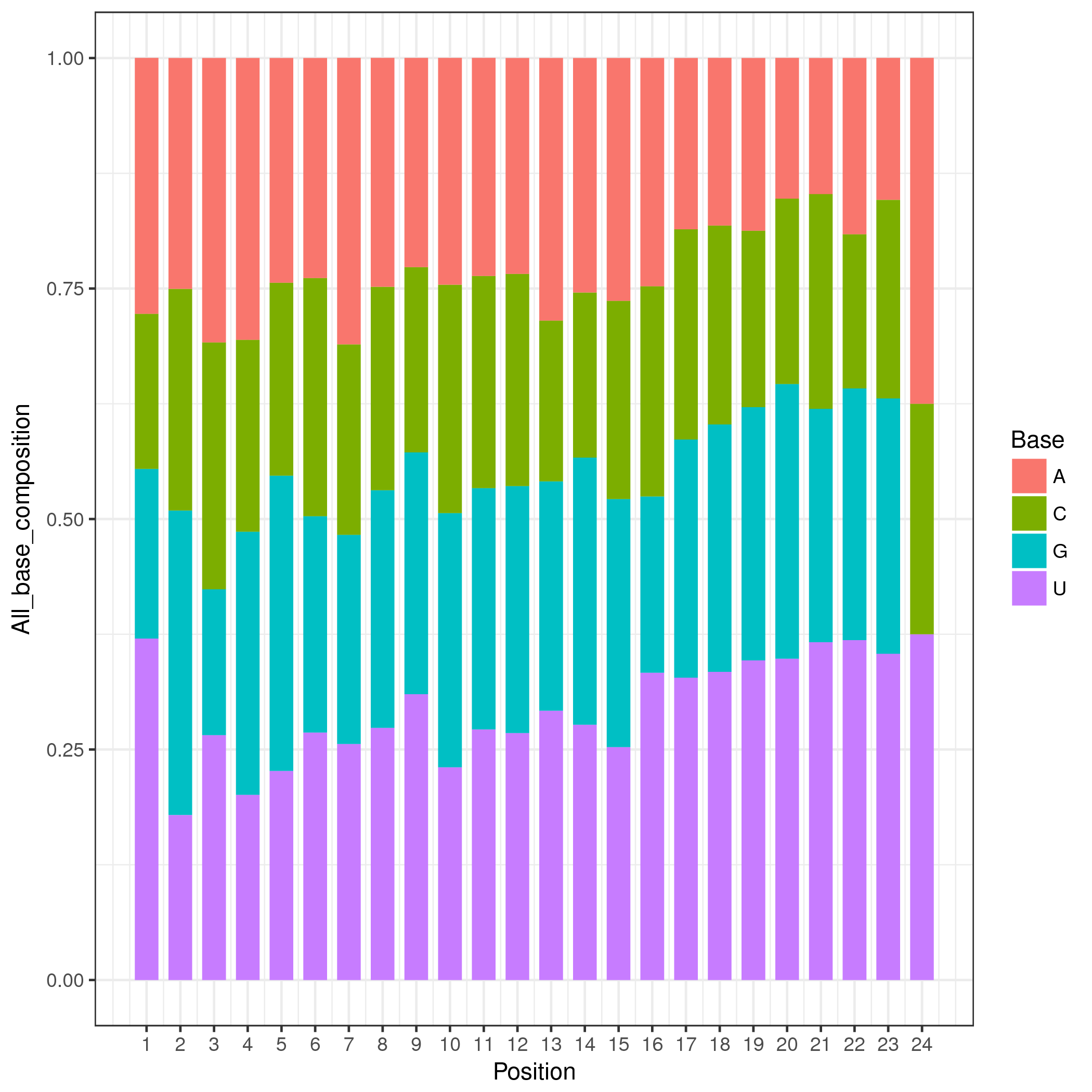

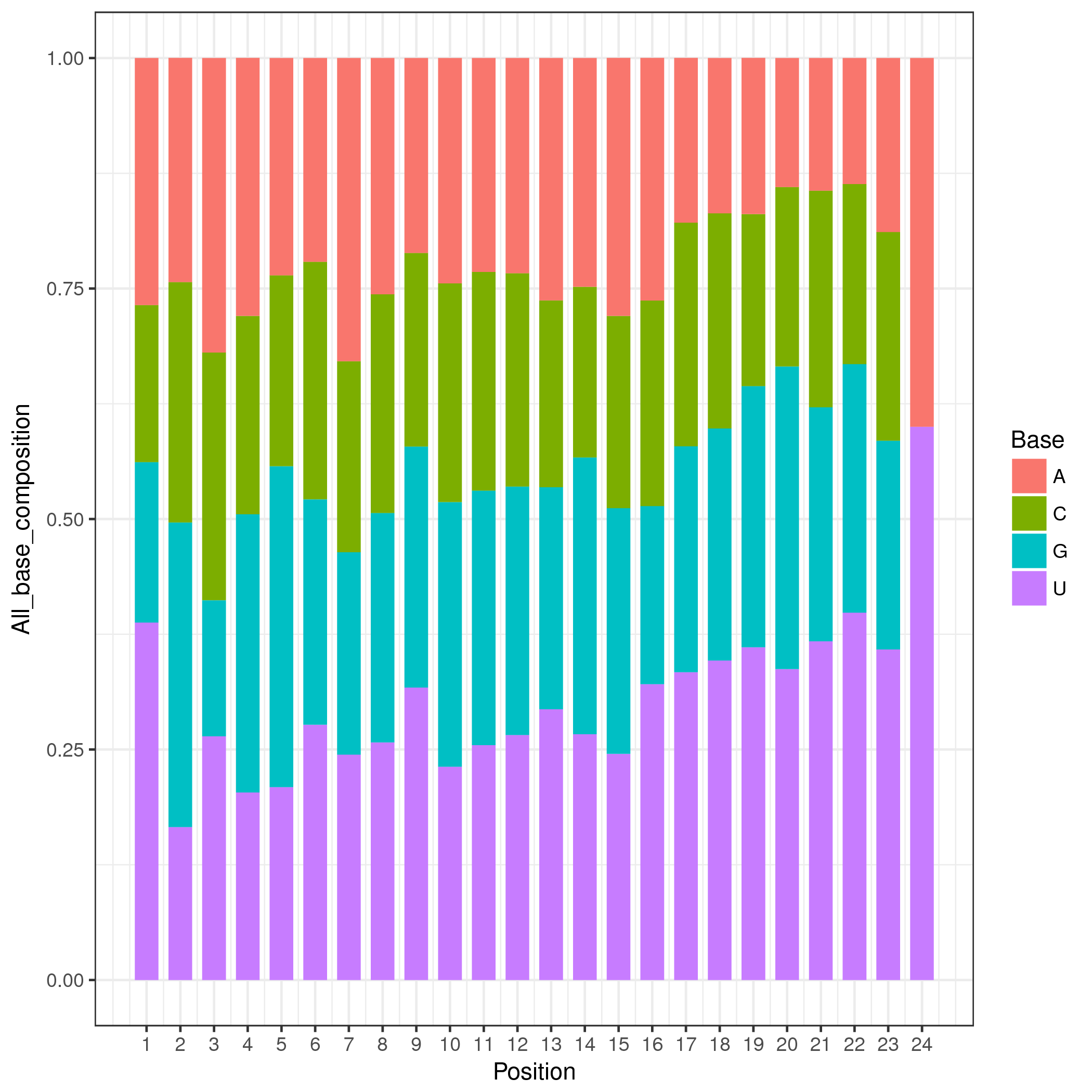

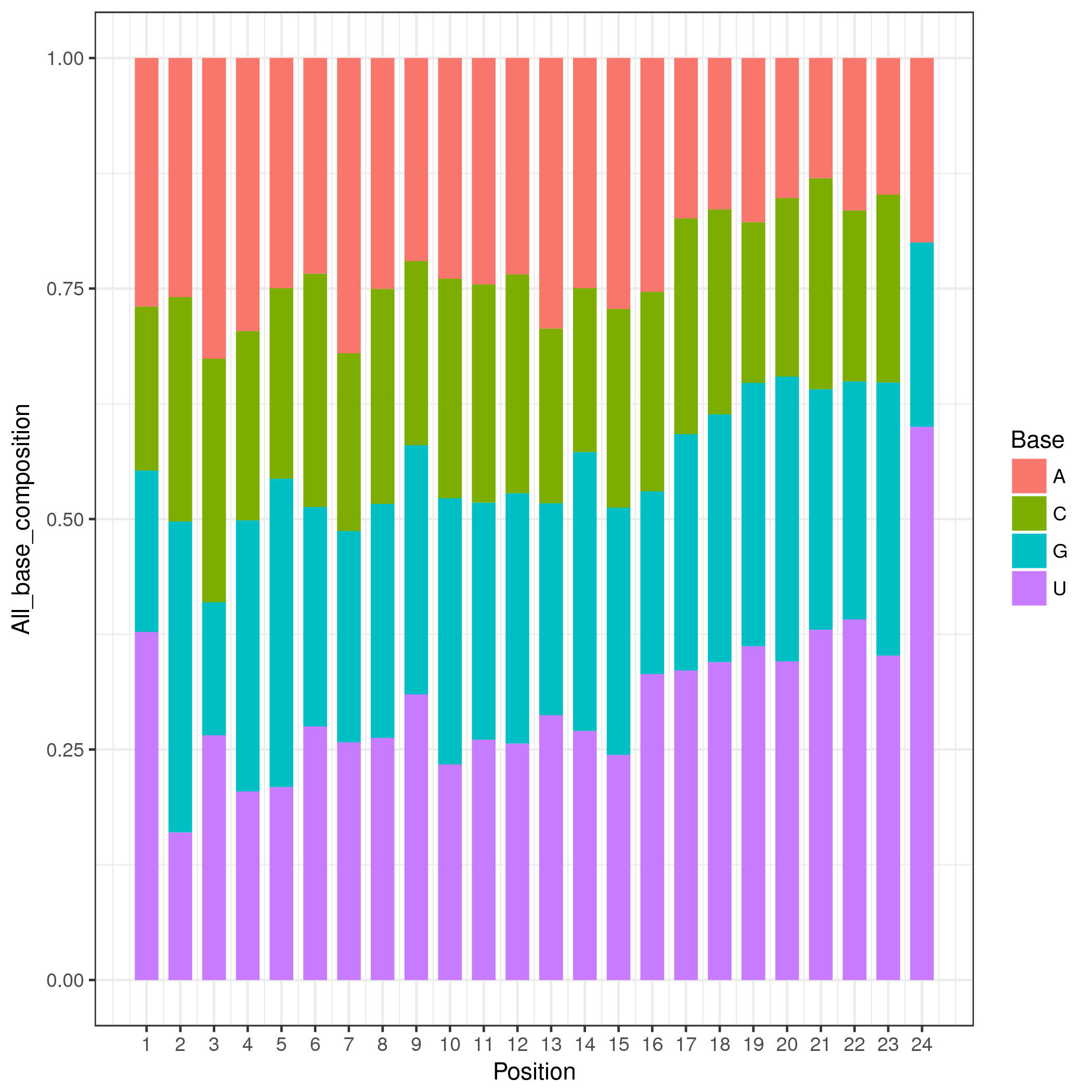

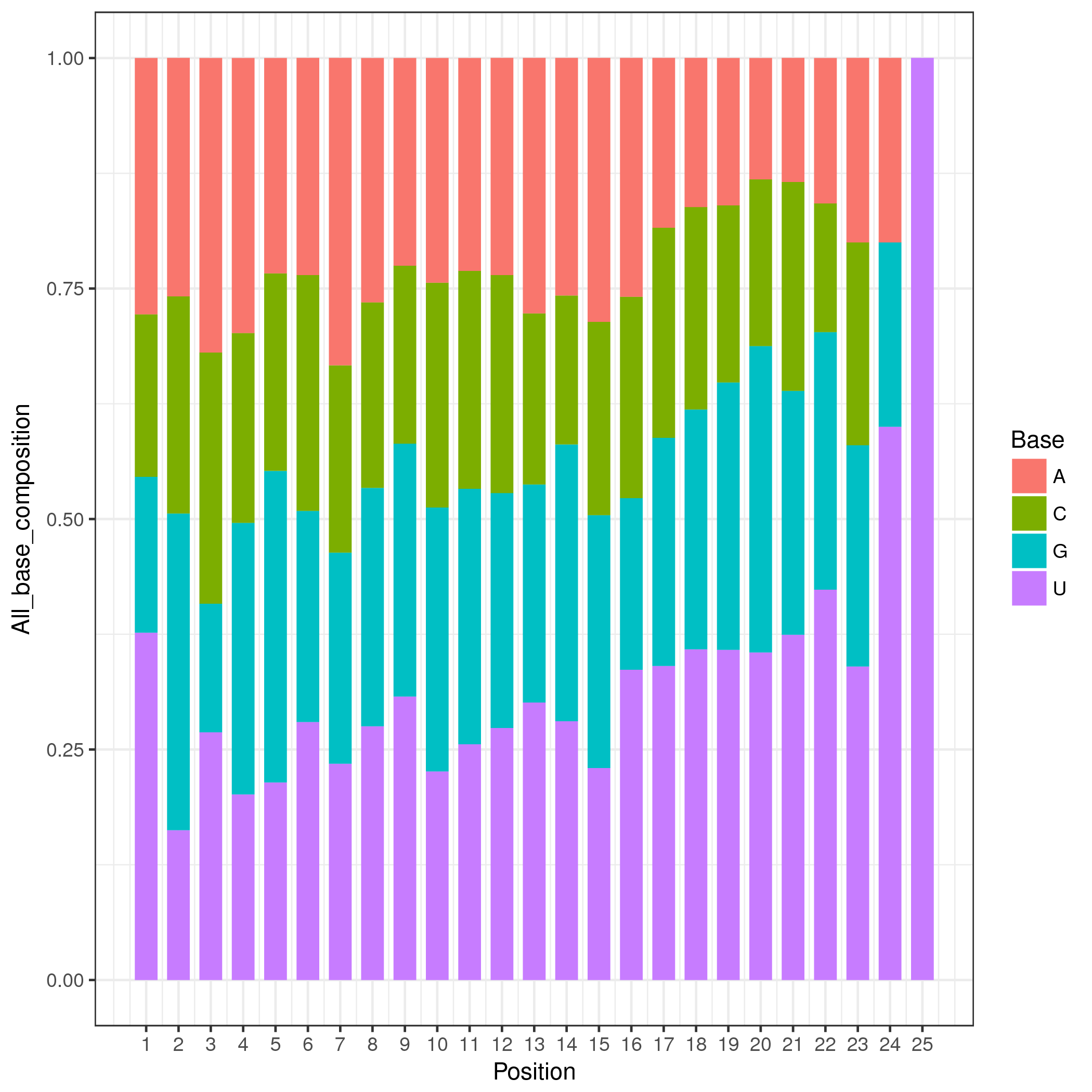

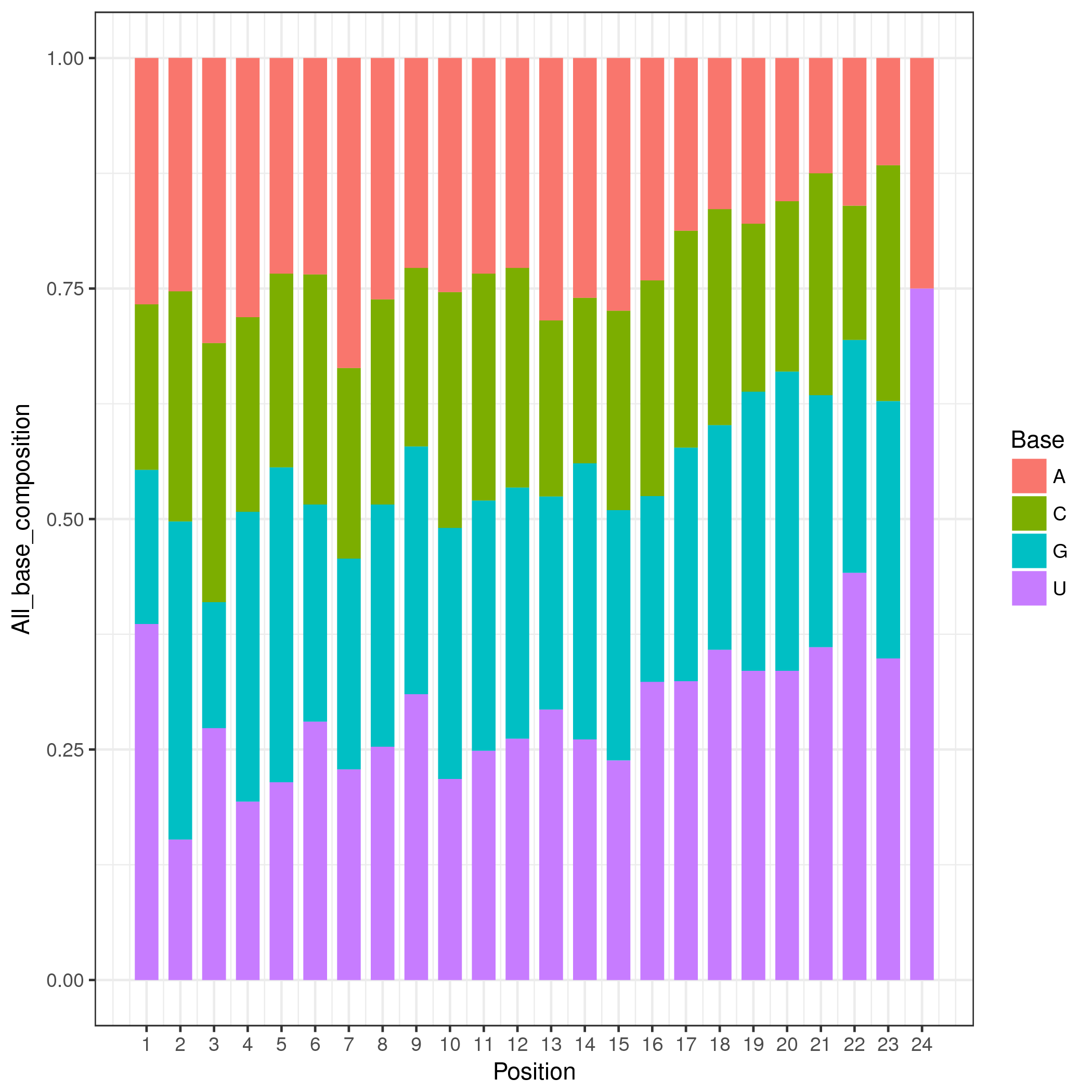

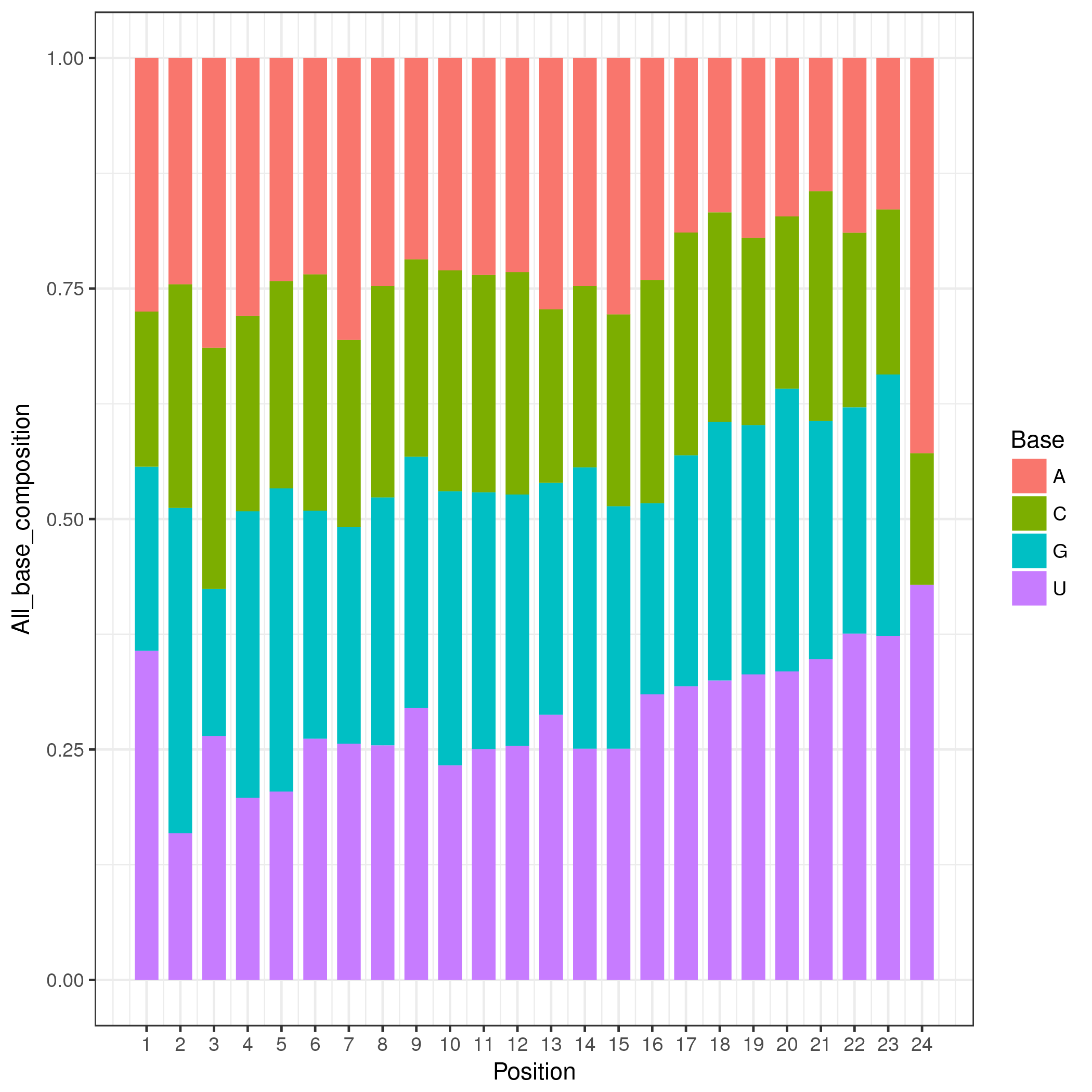

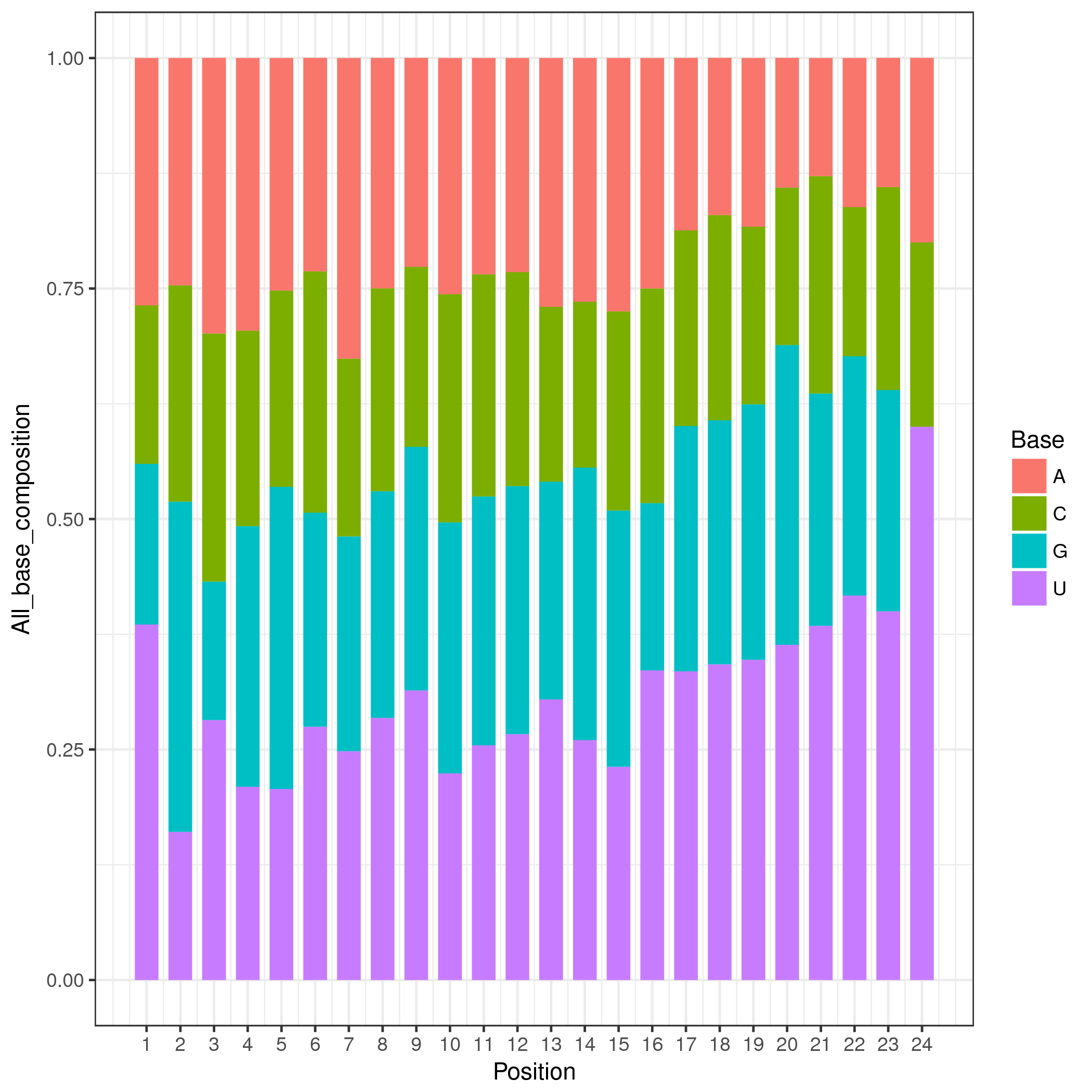


CCA

before surgery

CCA

after surgery

Normal

GBC

before surgery

GBC

after surgery

Figure. S2. Nucleotides preference of exosomal small RNAs of normal individuals, CCA and GBC patients before and after surgeries


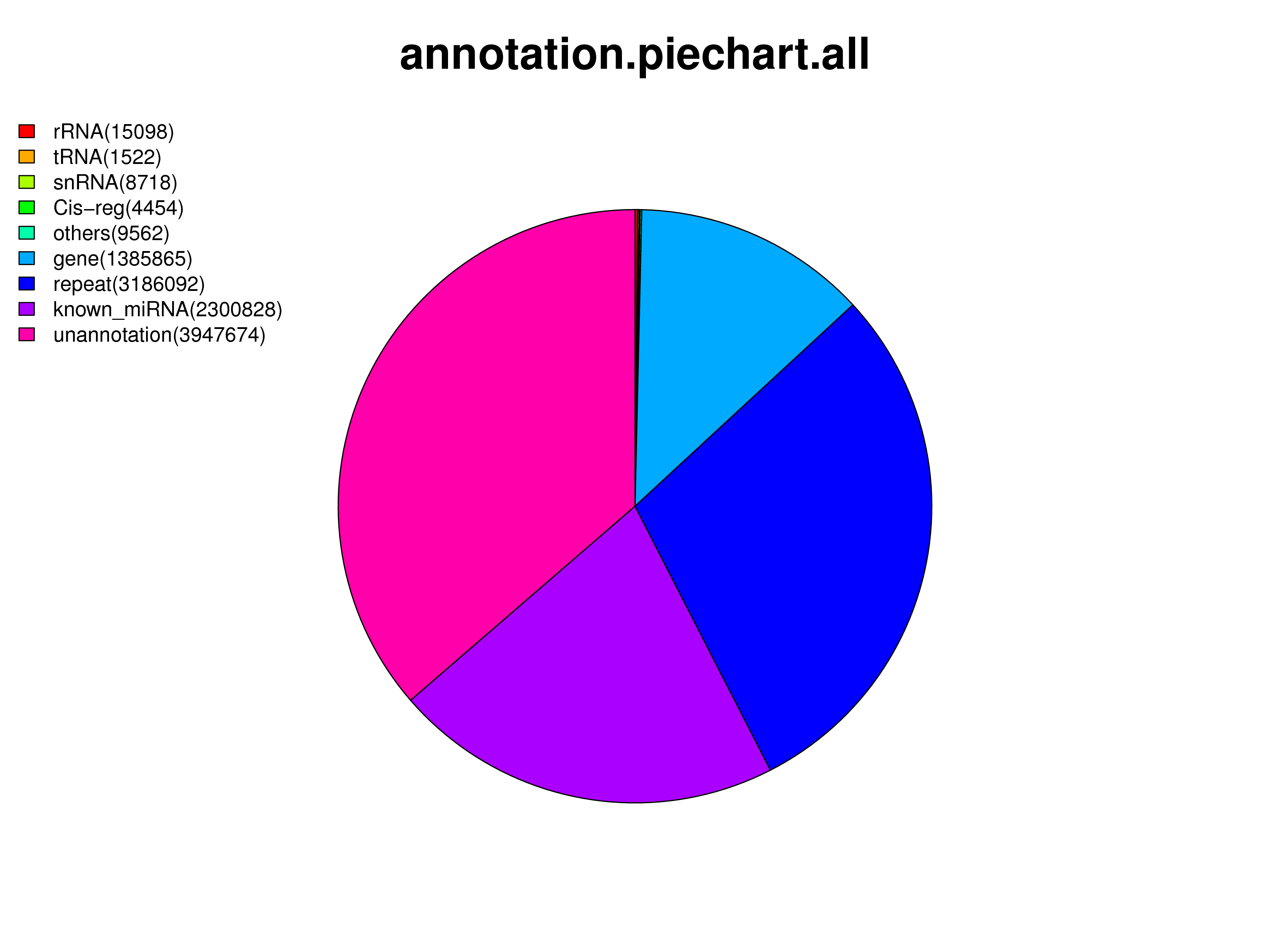

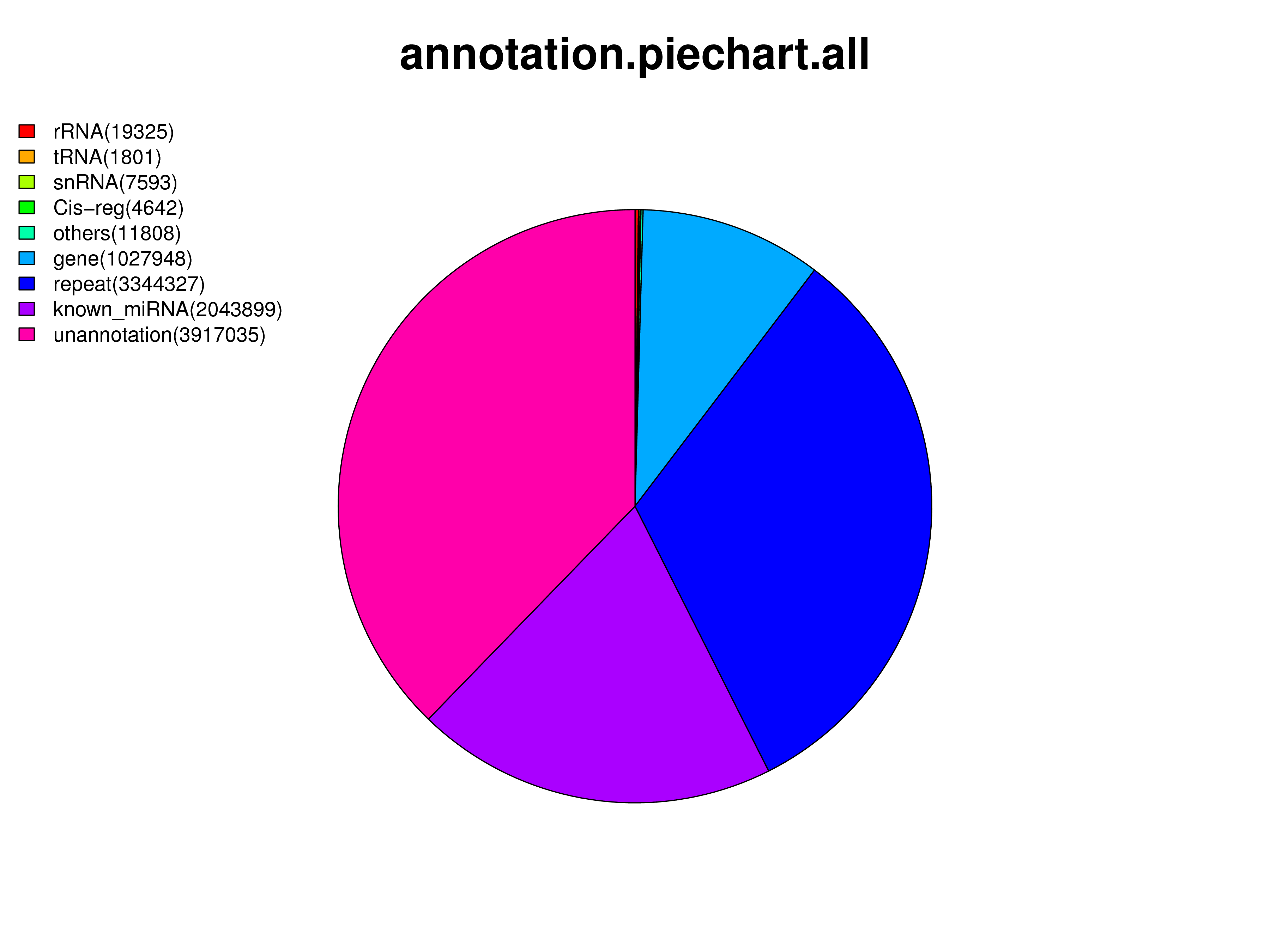

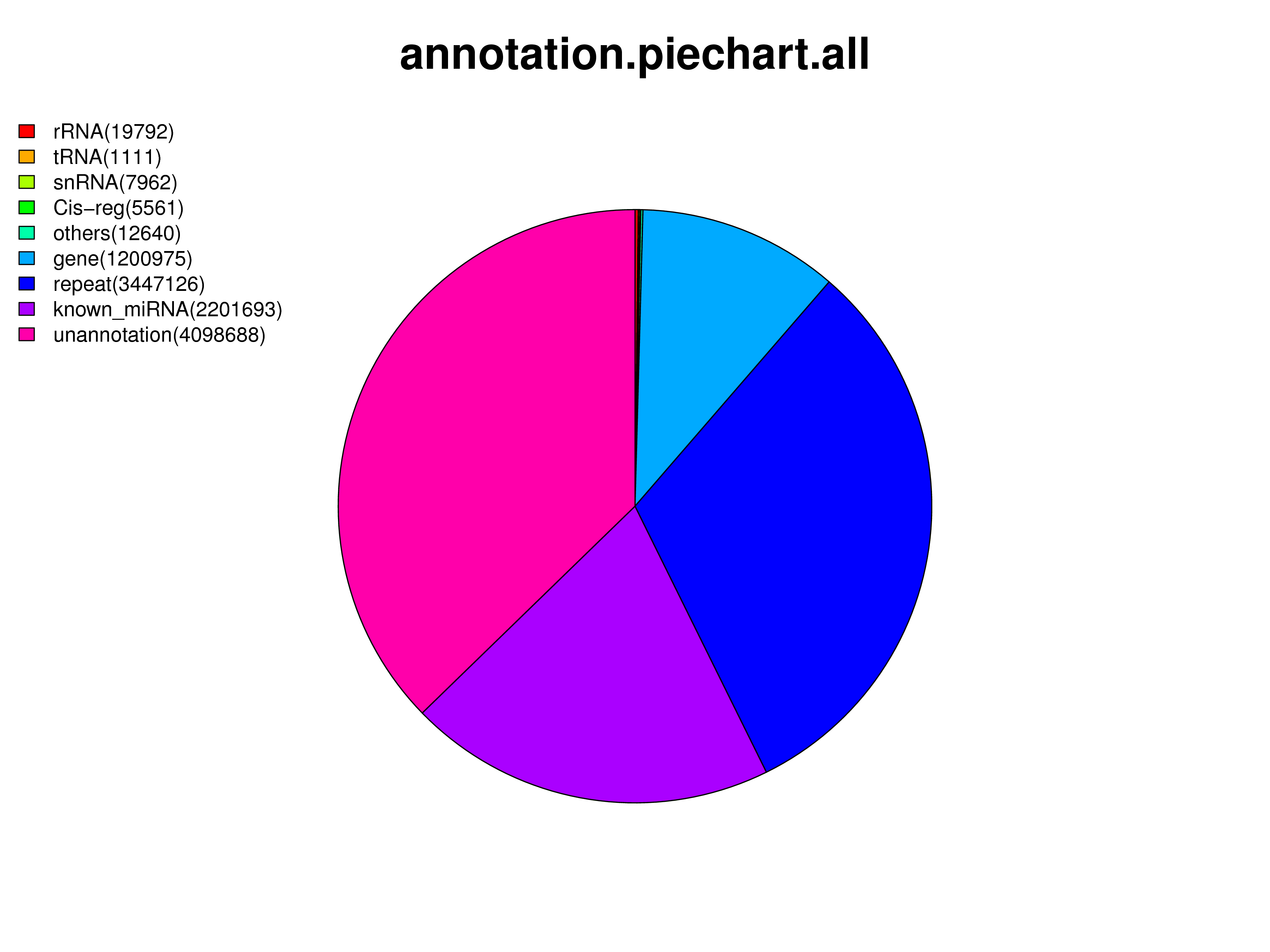

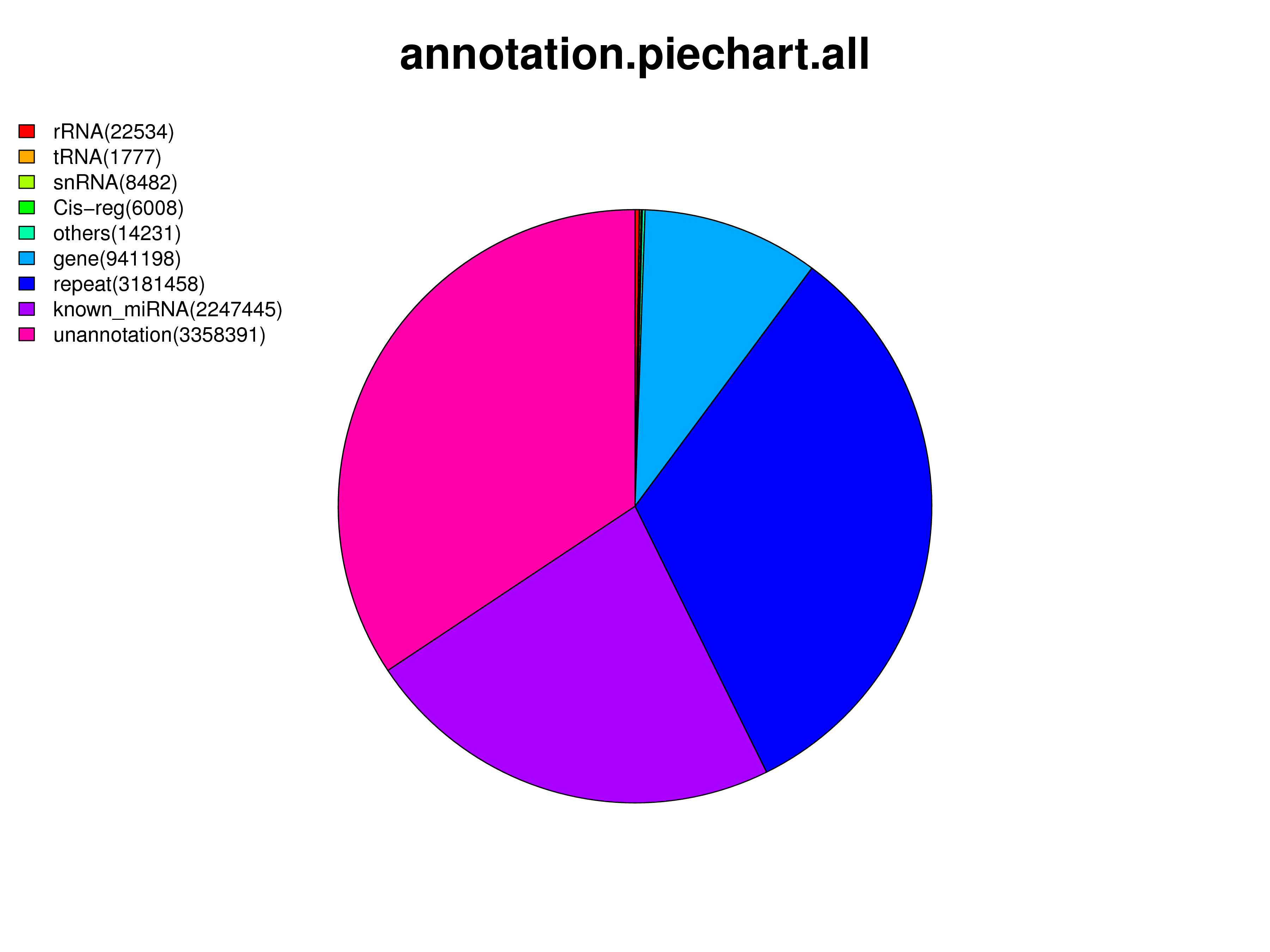

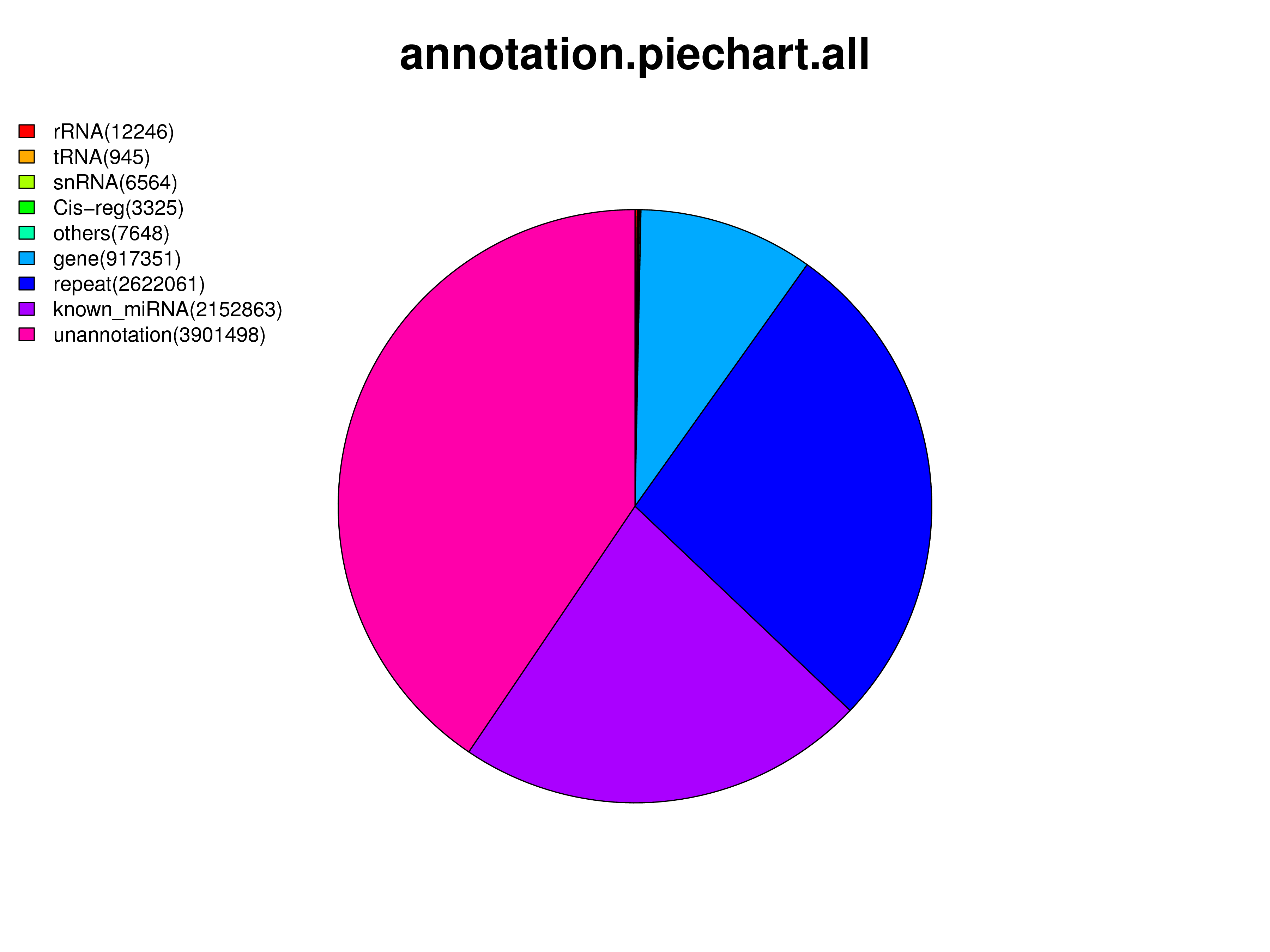

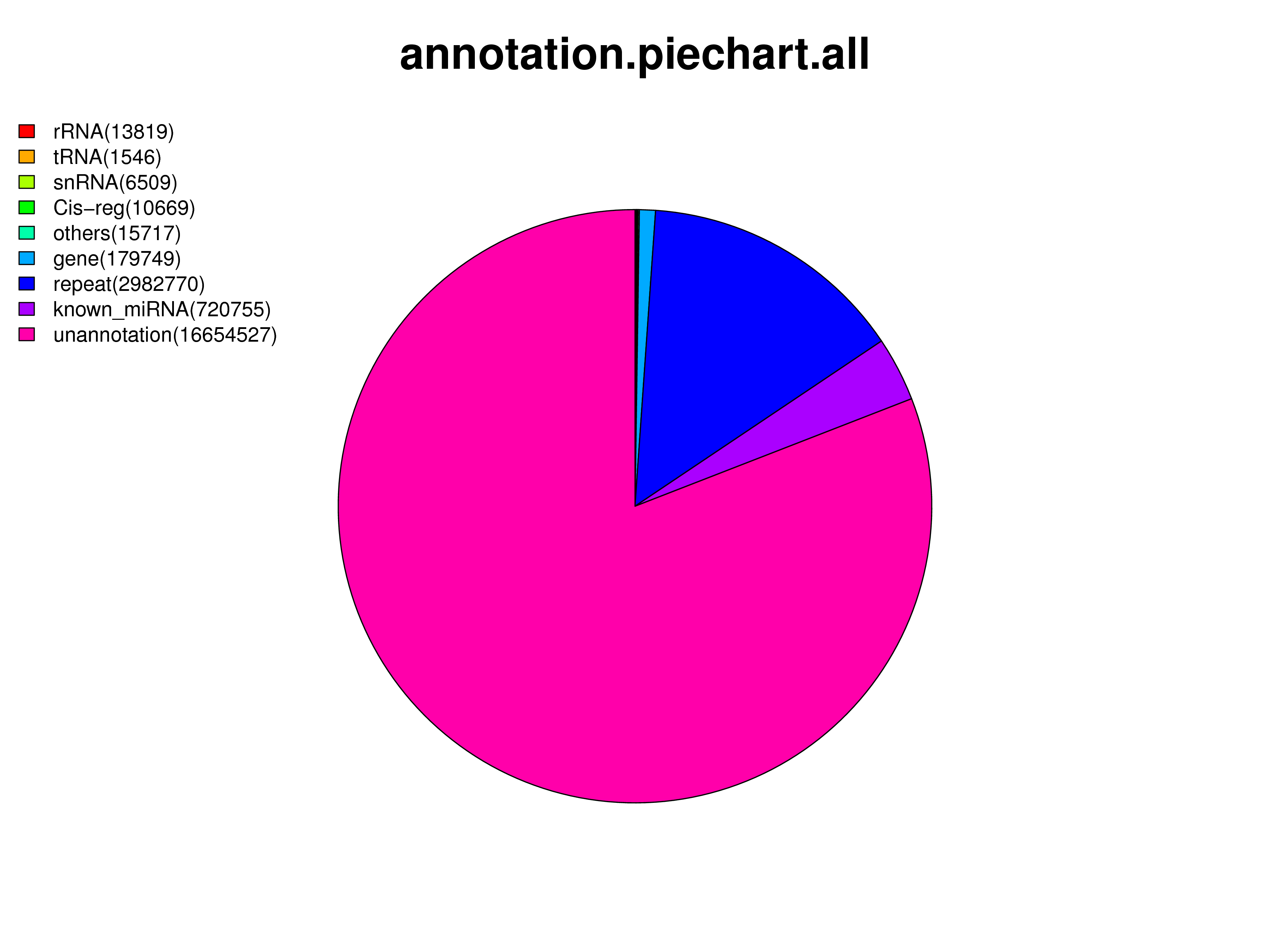

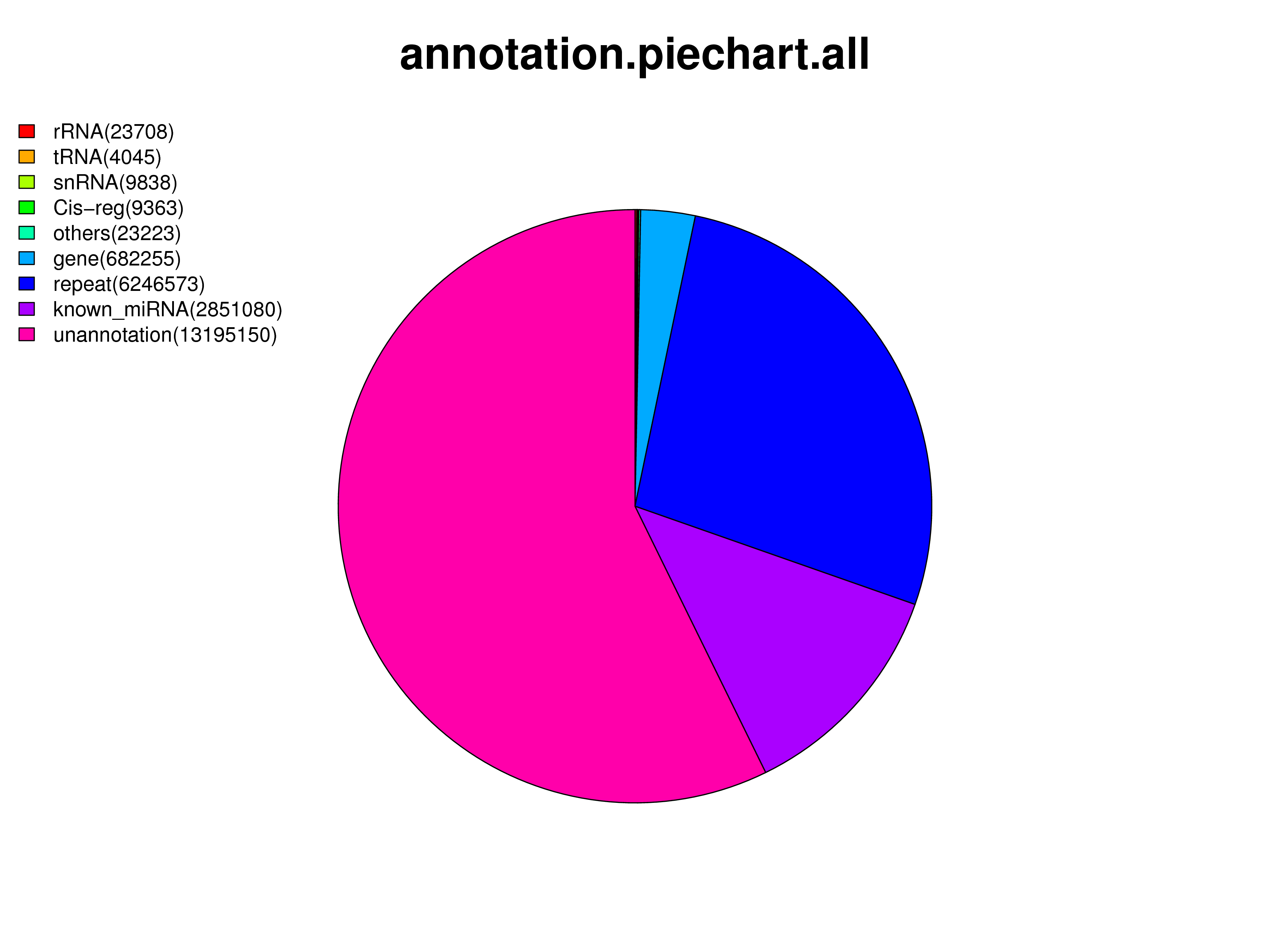

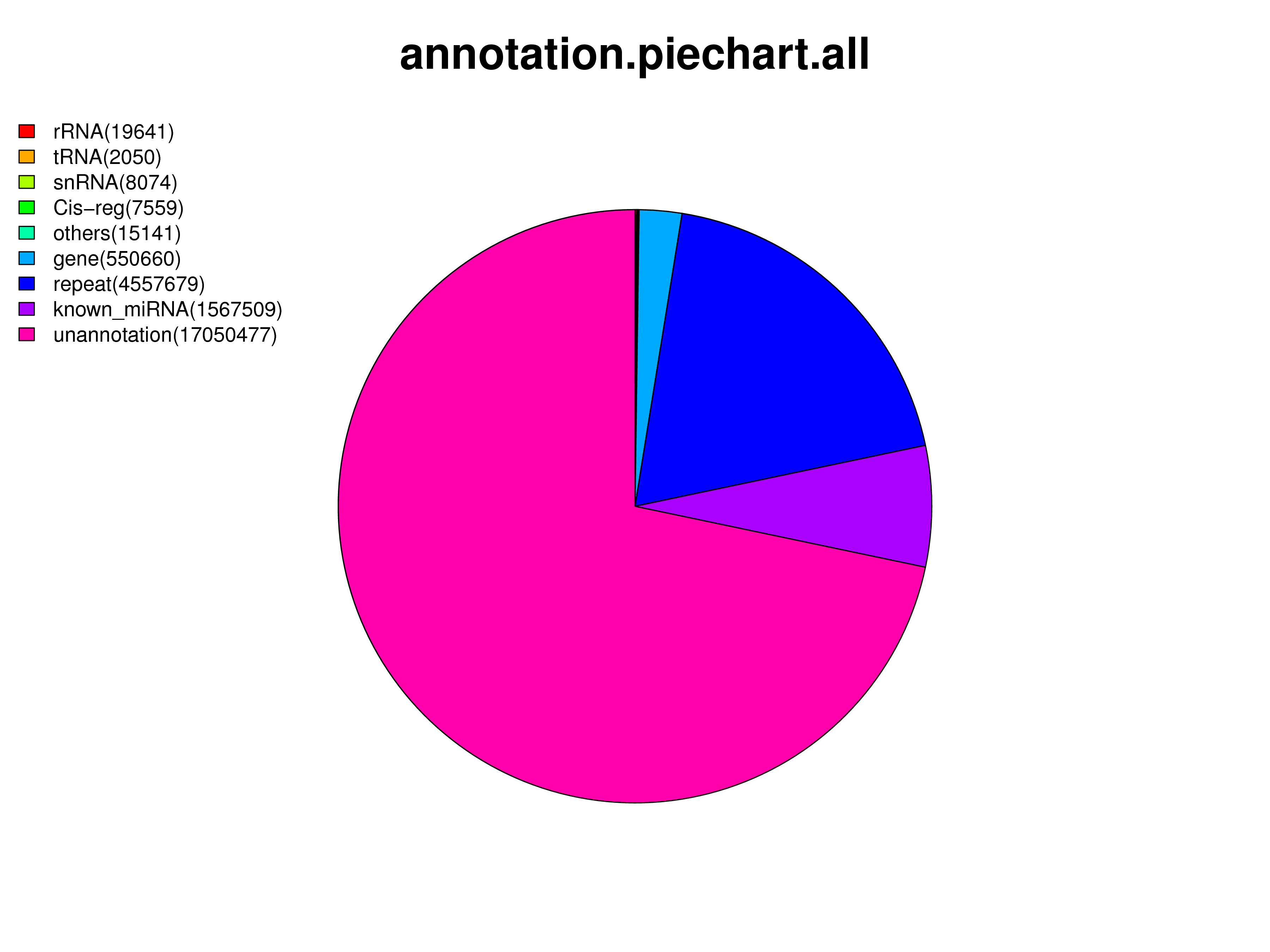

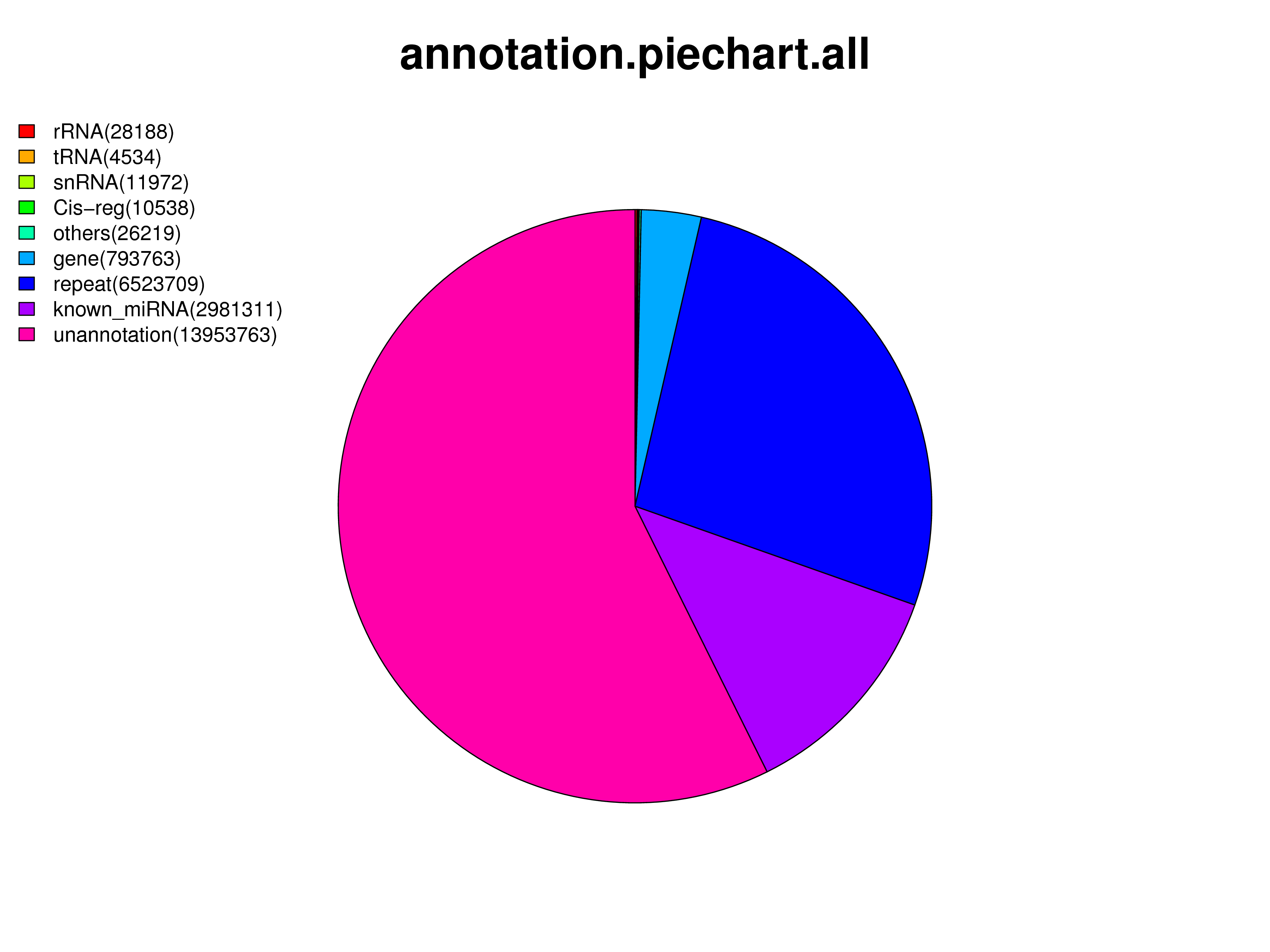

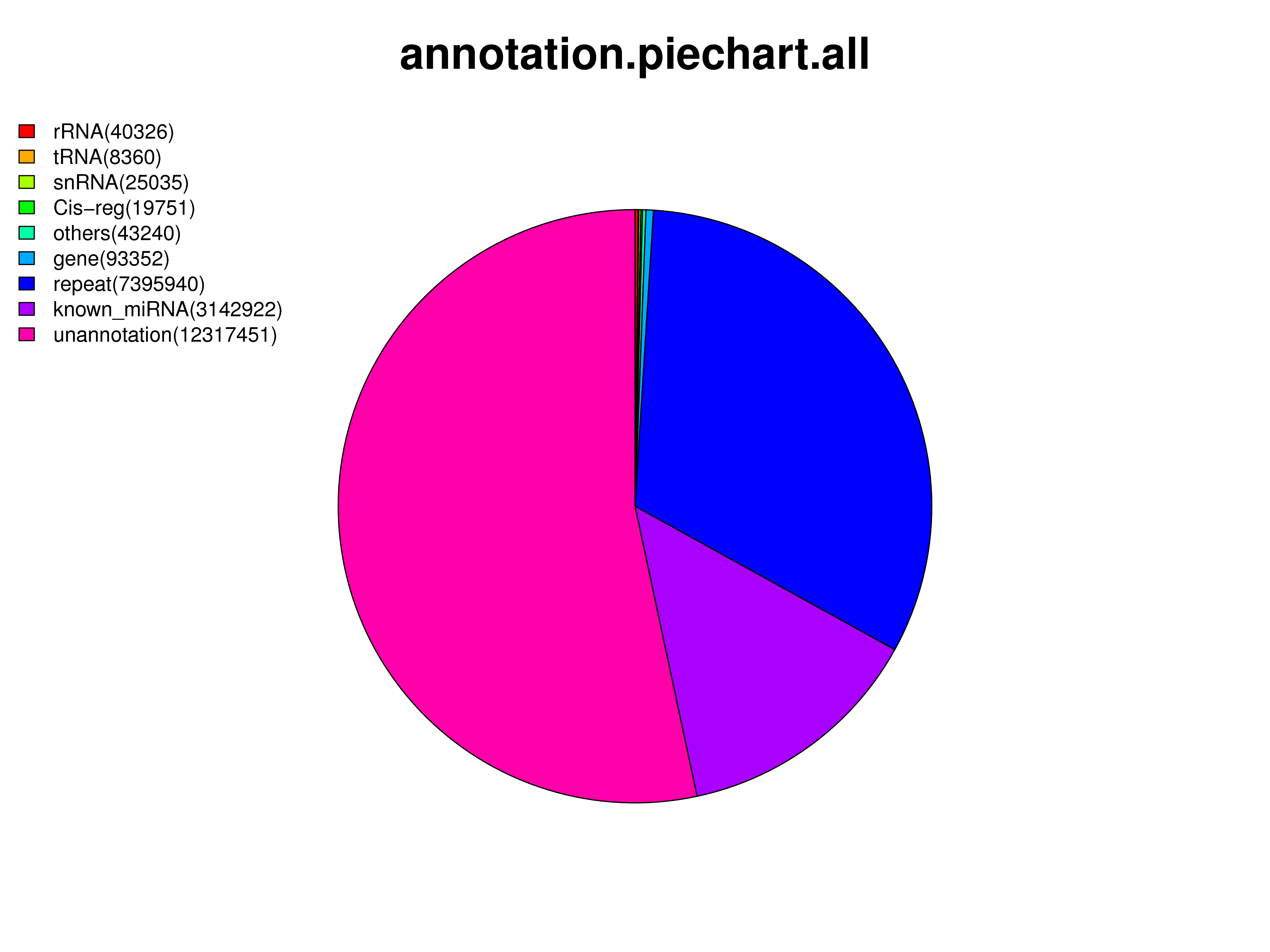

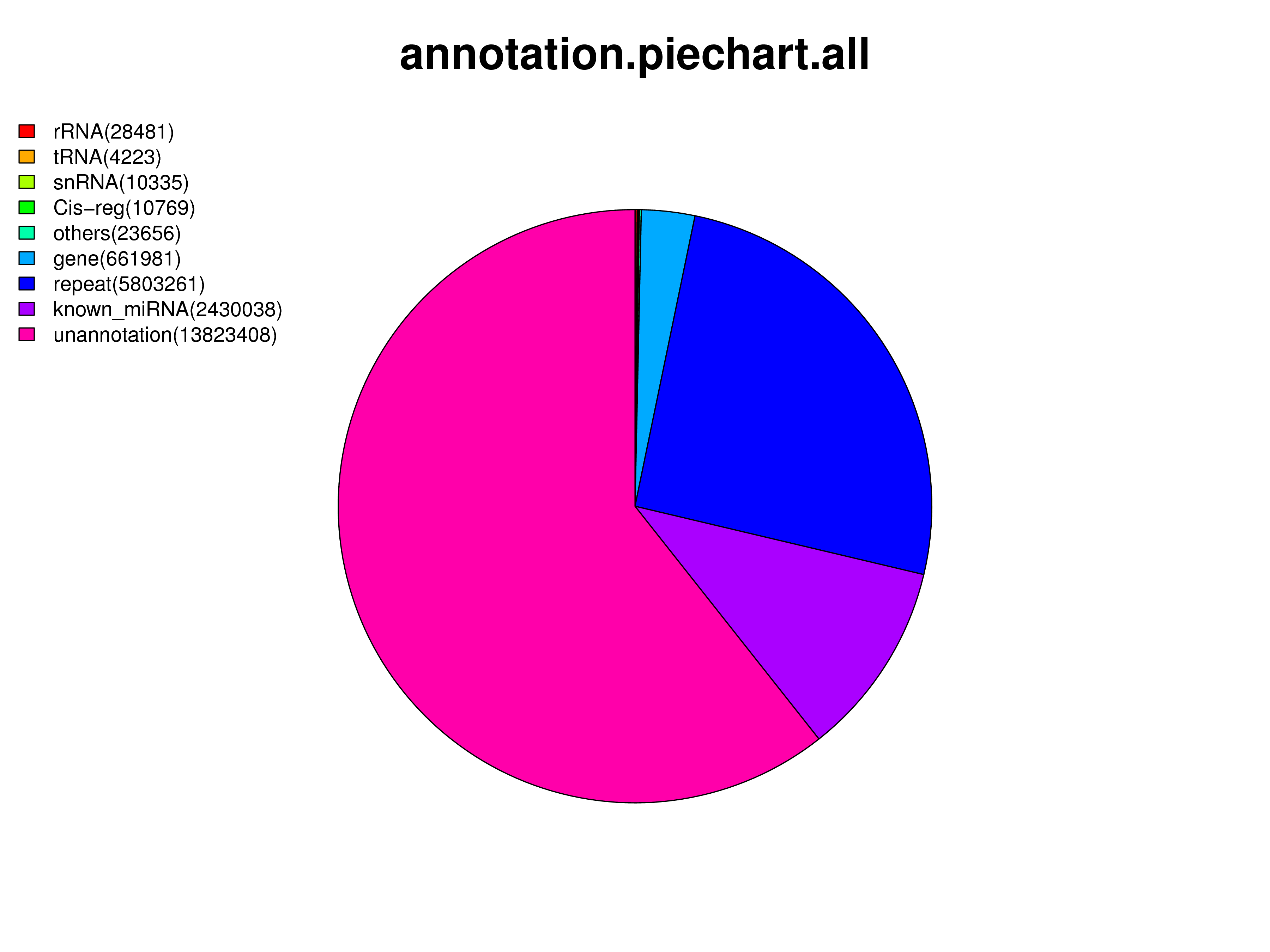

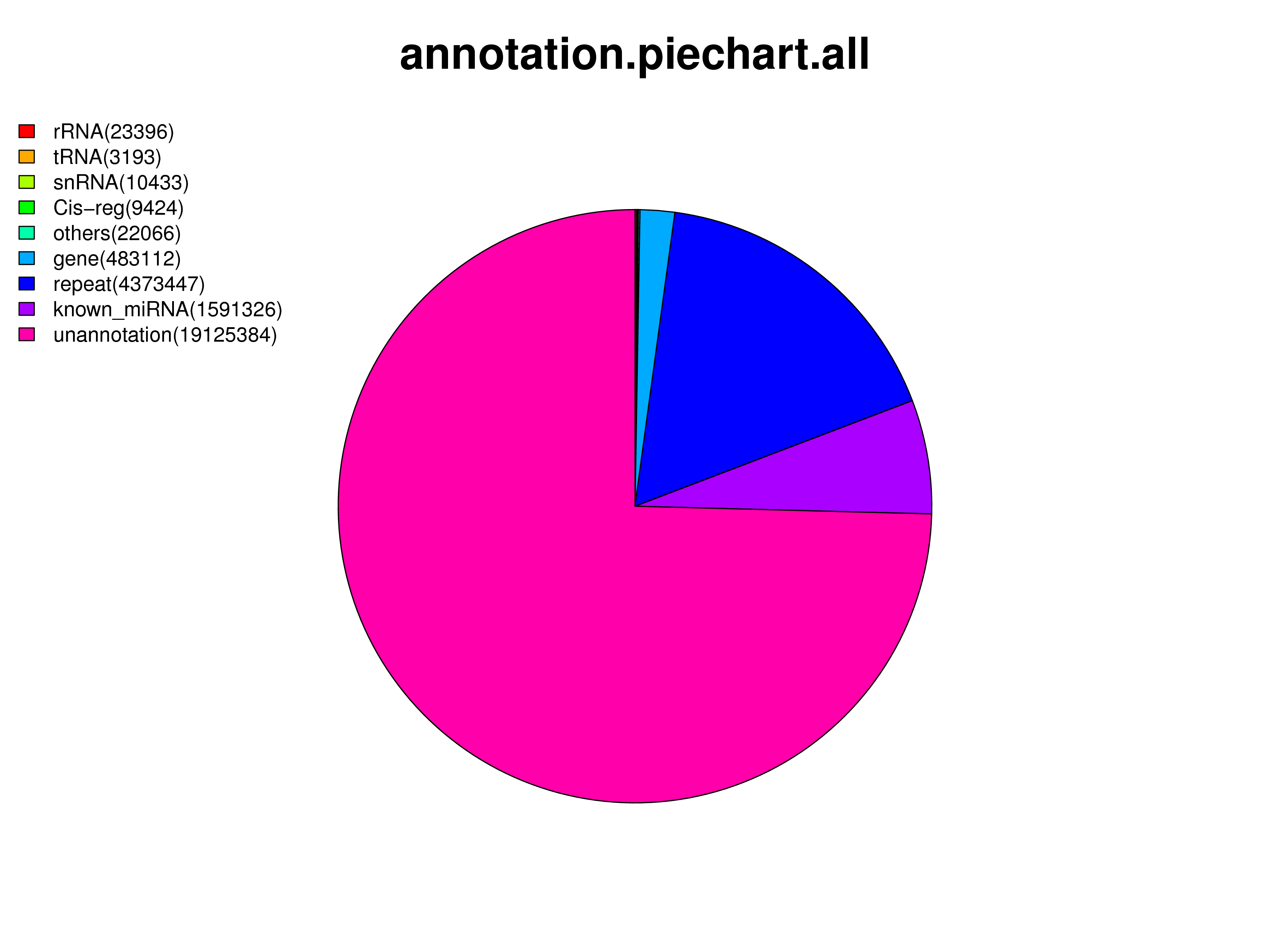

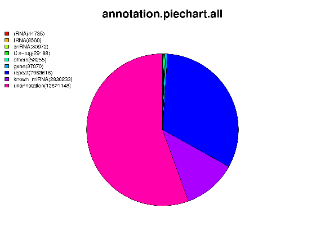

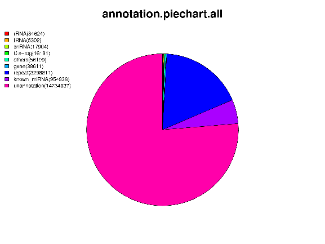

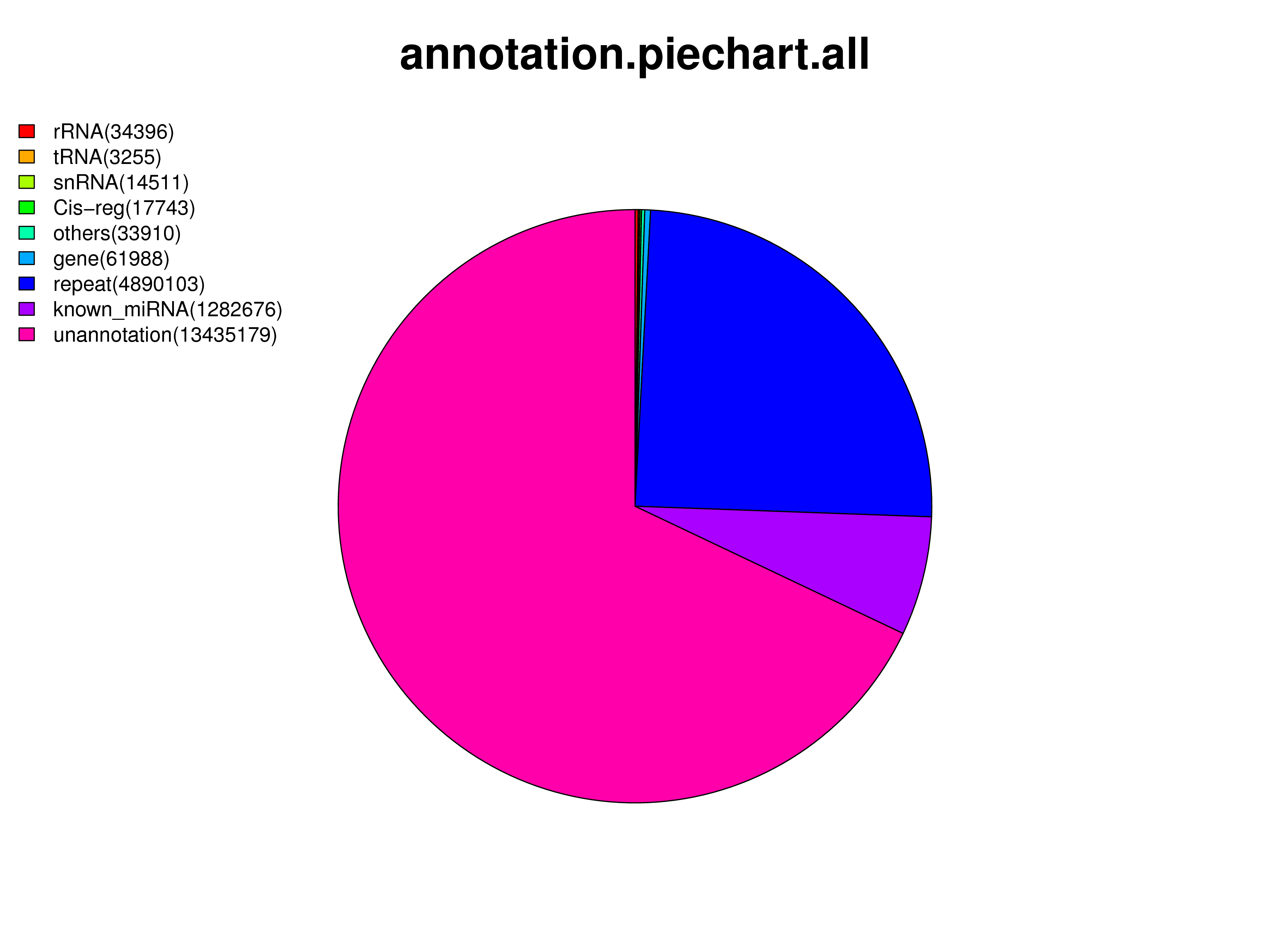

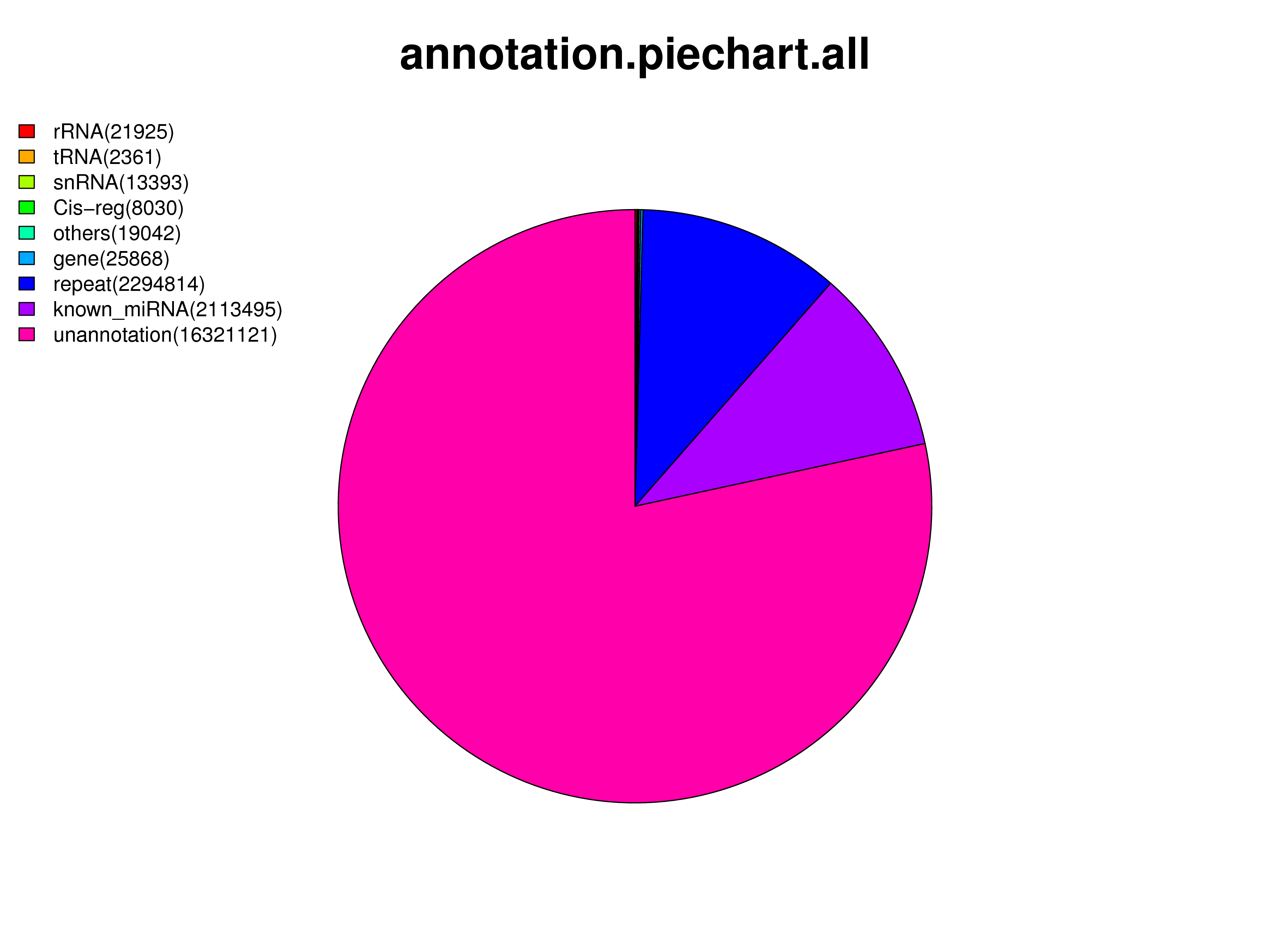

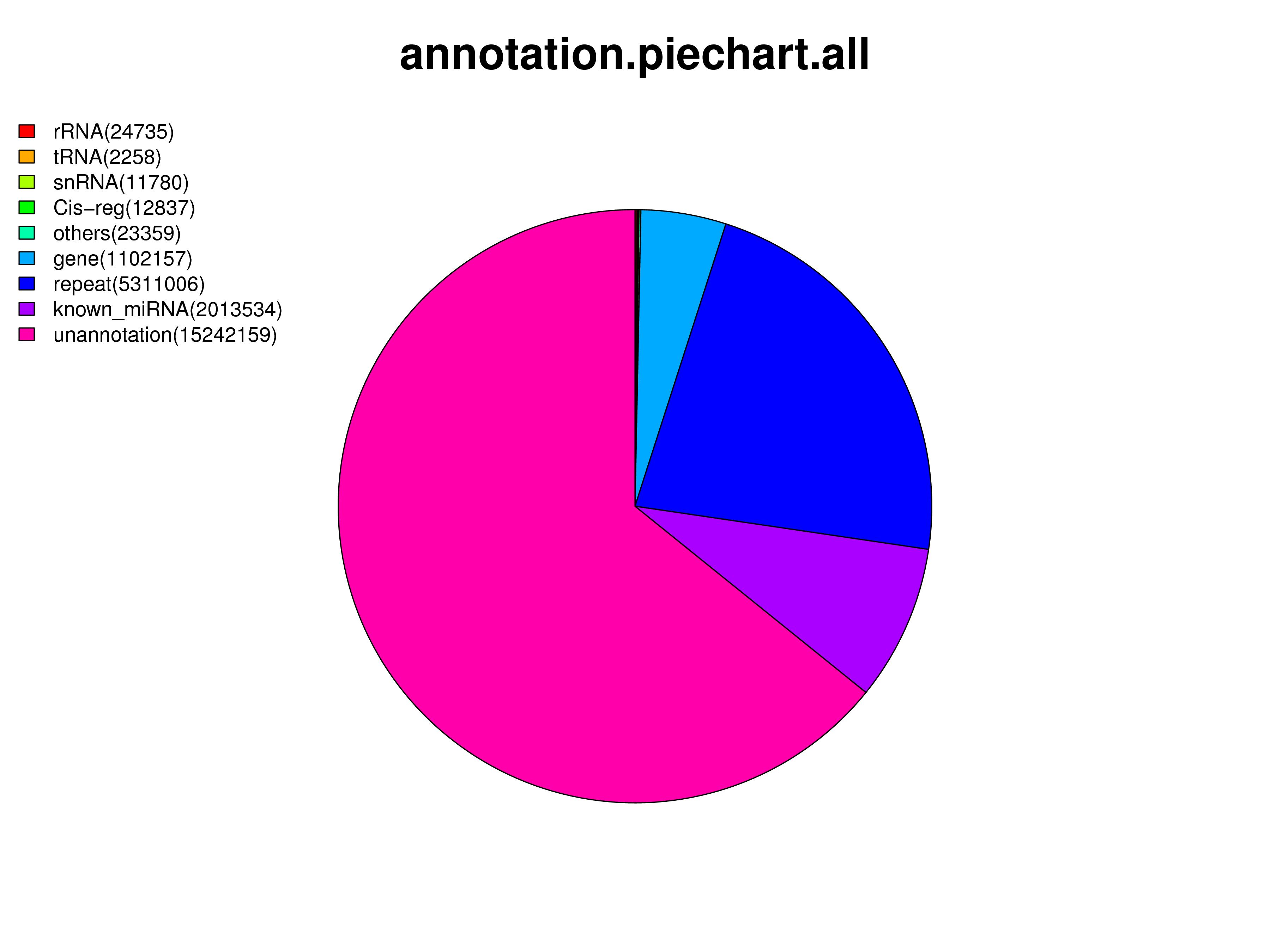


CCA

before surgery

CCA

after surgery

Normal

GBC

before surgery

GBC

after surgery


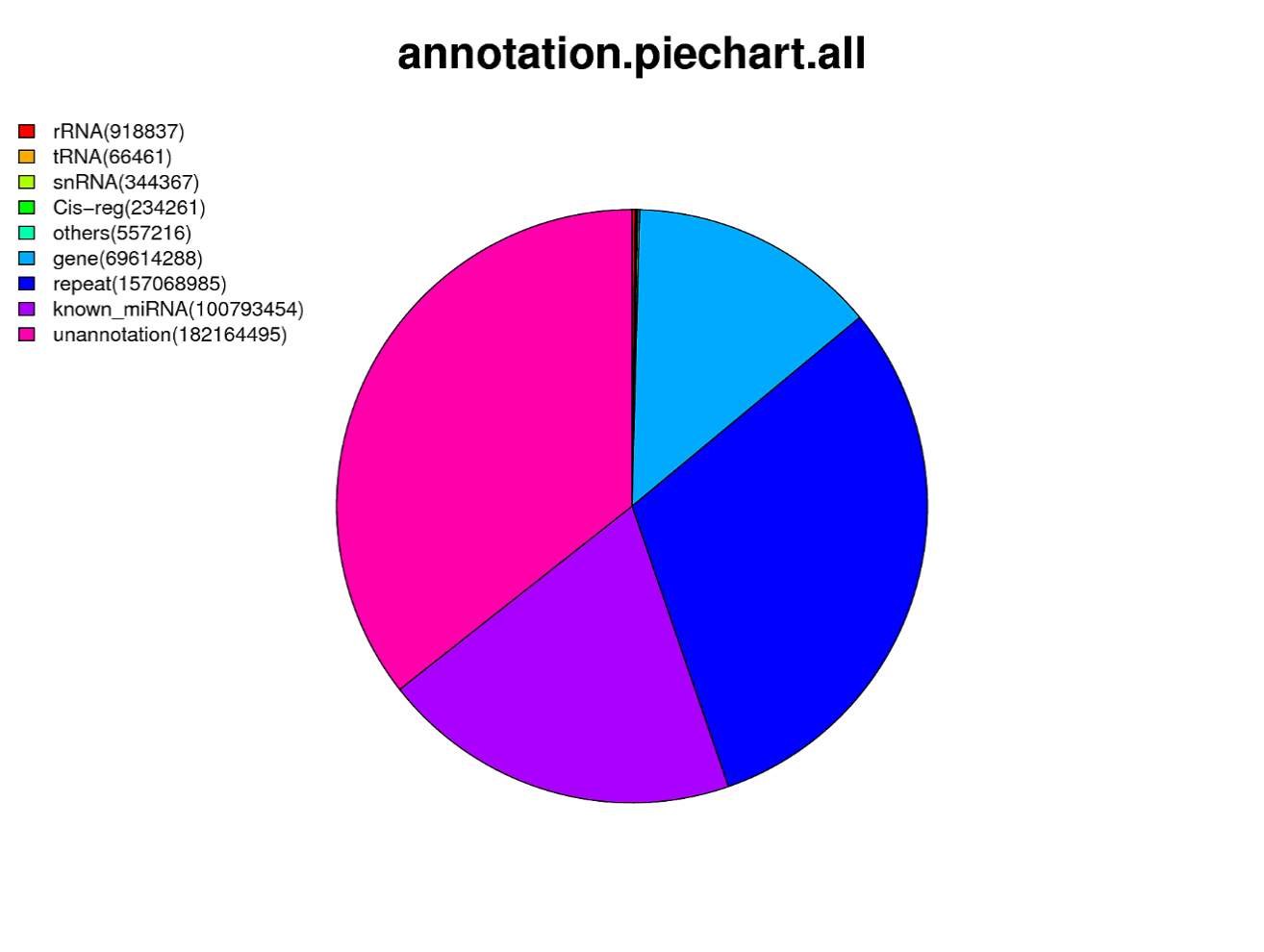

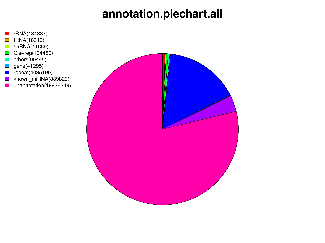

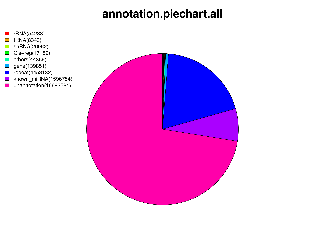

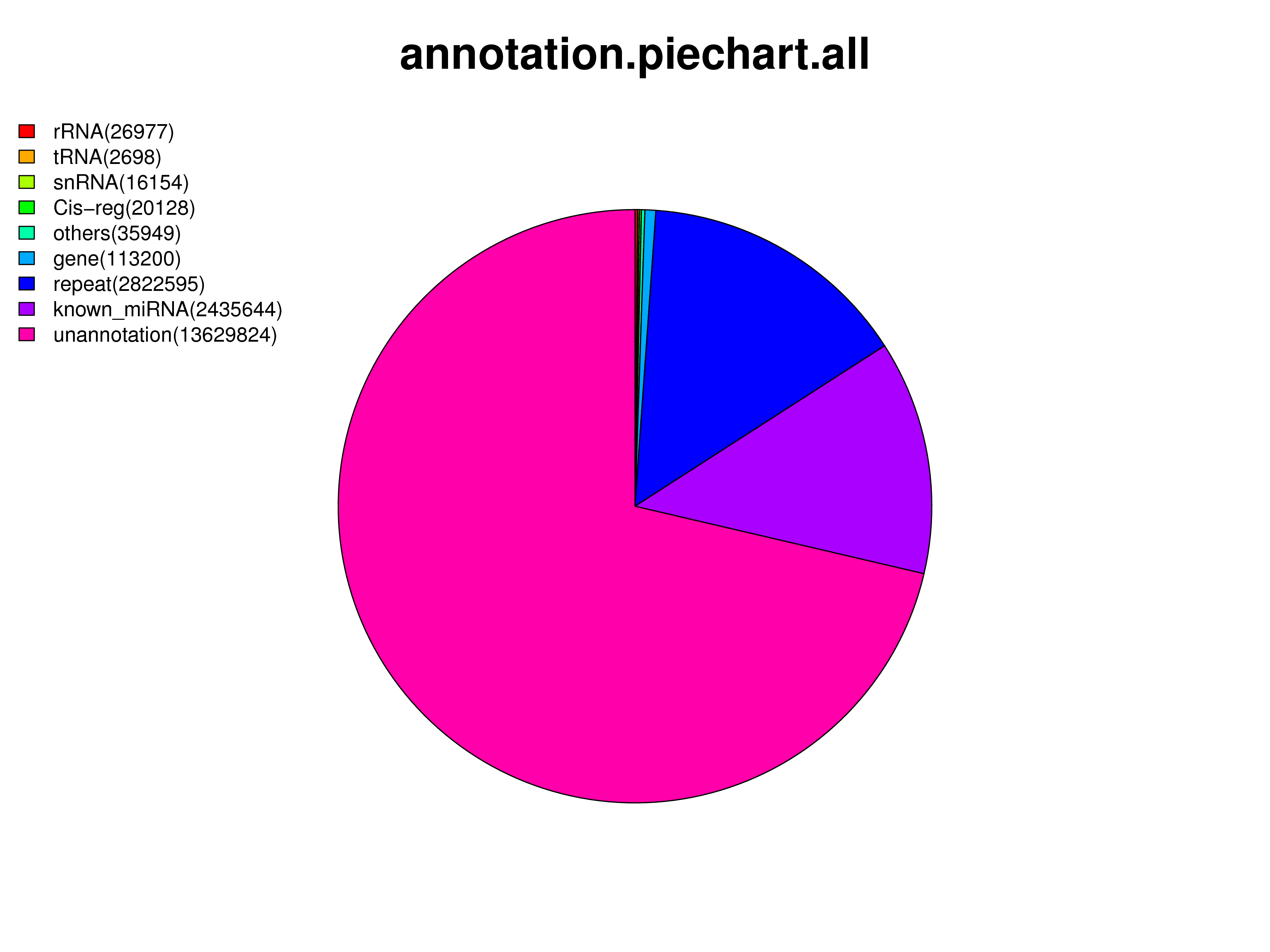

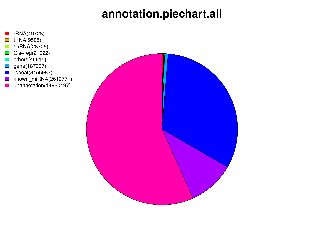

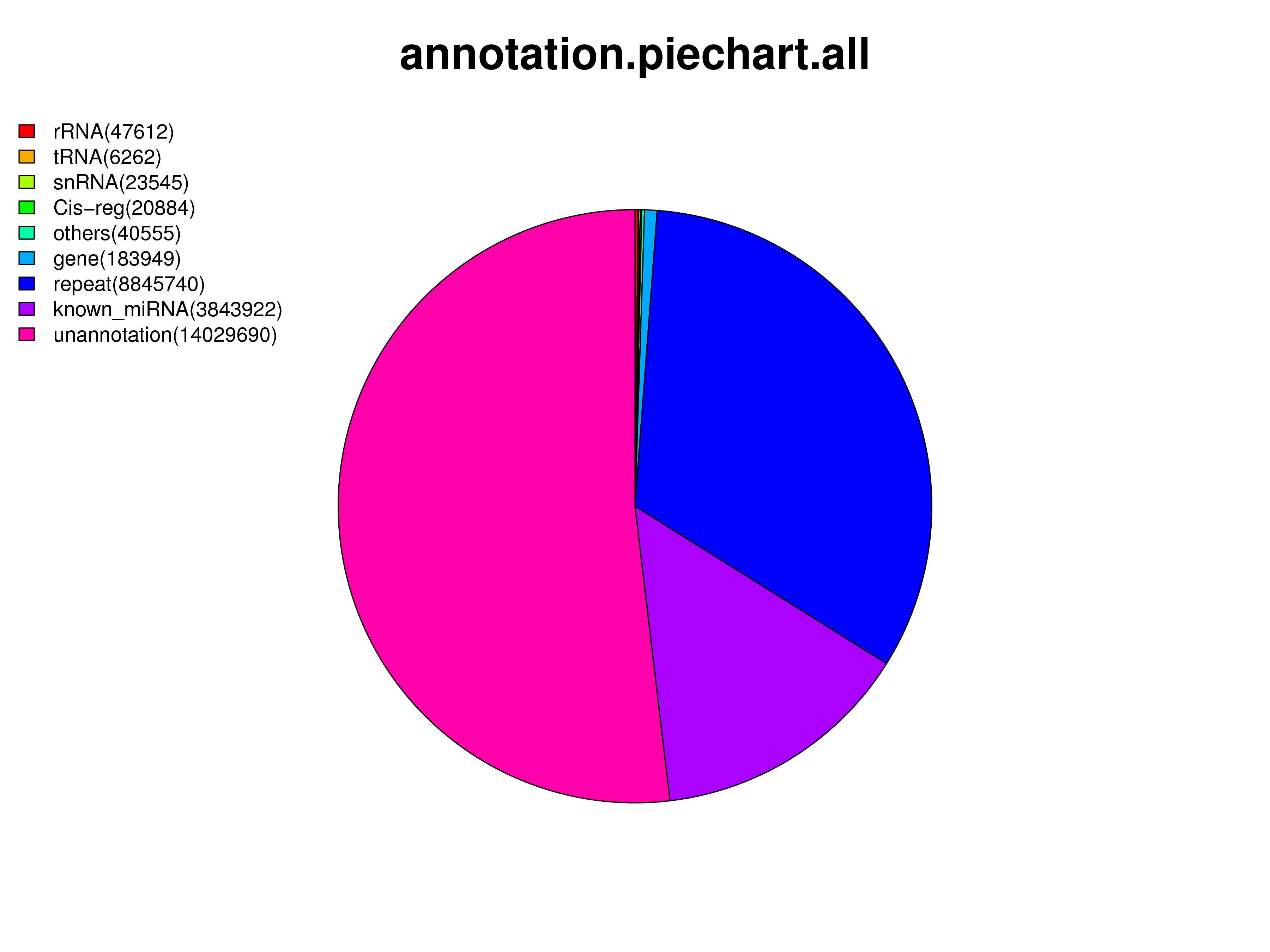

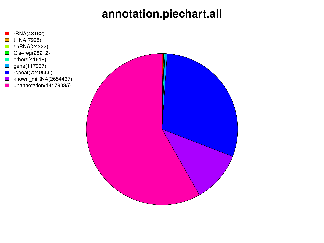


Figure. S3. Annotations of exosomal small RNAs of normal individuals, CCA and GBC patients before and after surgeries


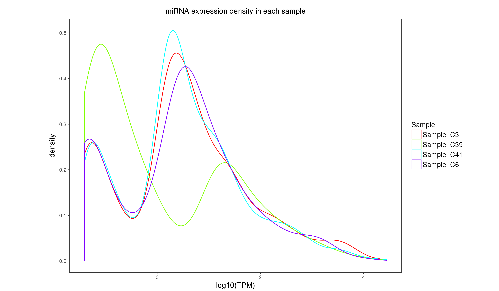

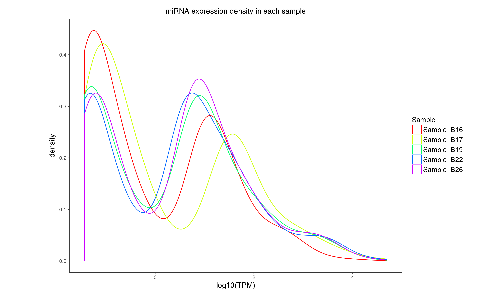


Cholangiocarcinoma

Gallbladder carcinoma

Normal control

**a**

**b**

**c**


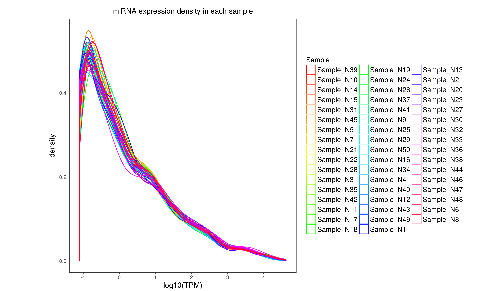


Figure. S4. Exosomal miRNA expression density of normal individuals, CCA and GBC patients

**a** Exosomal miRNA expression density of normal individuals.

**b** Exosomal miRNA expression density of CCA patients.

**c** Exosomal miRNA expression density of GBC patients.


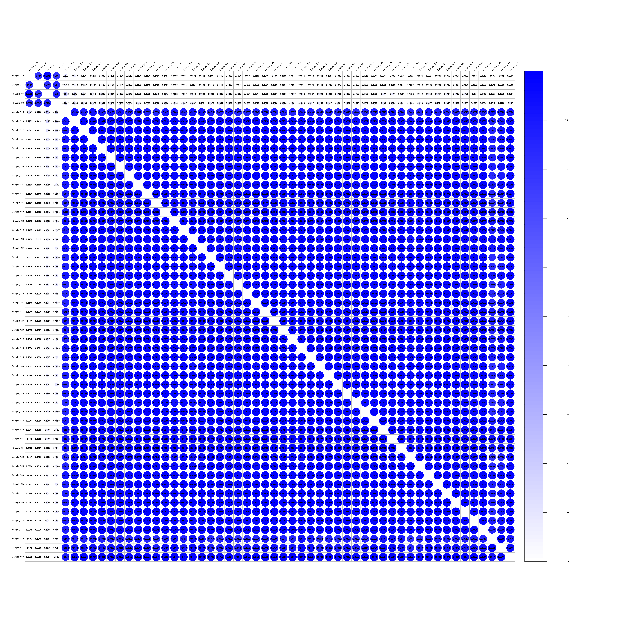

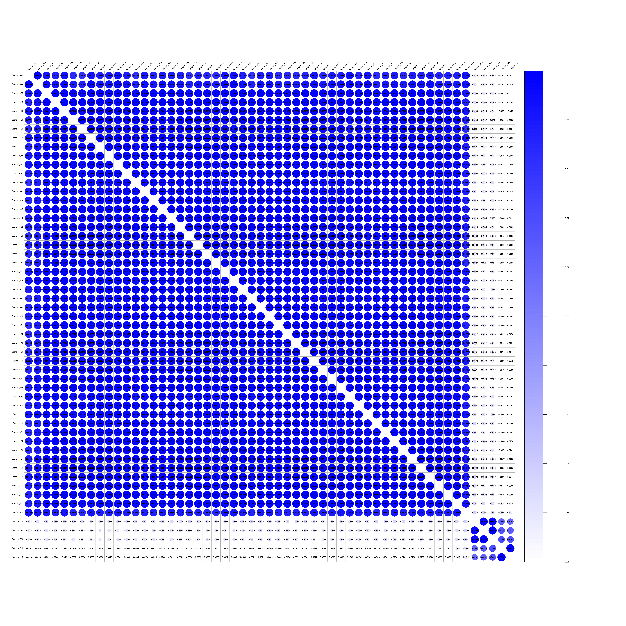


GBC

CCA

Normal

Normal

**a**

**b**

Figure. S5. . Expression correlation of normal individuals, CCA and GBC patients derived exosomes

**a** Expression correlation of normal individuals and CCA patients derived exosomes.

**b** Expression correlation of normal individuals and GBC patients derived exosomes.


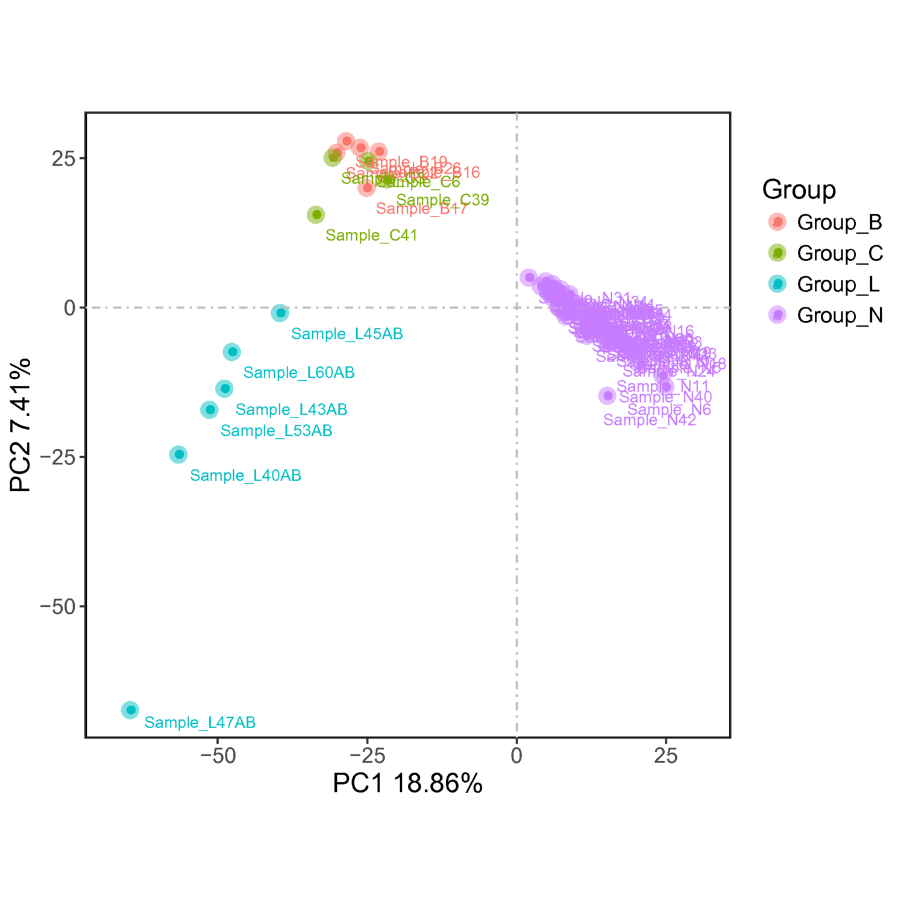


(CCA)

(GBC)

(Lung Cancer)

(Normal)

Figure. S6. Principal component analysis (PCA) of normal individuals, CCA and GBC patients derived exosomes

Cholangiocarcinoma

Gallbladder carcinoma

**a**

**b**


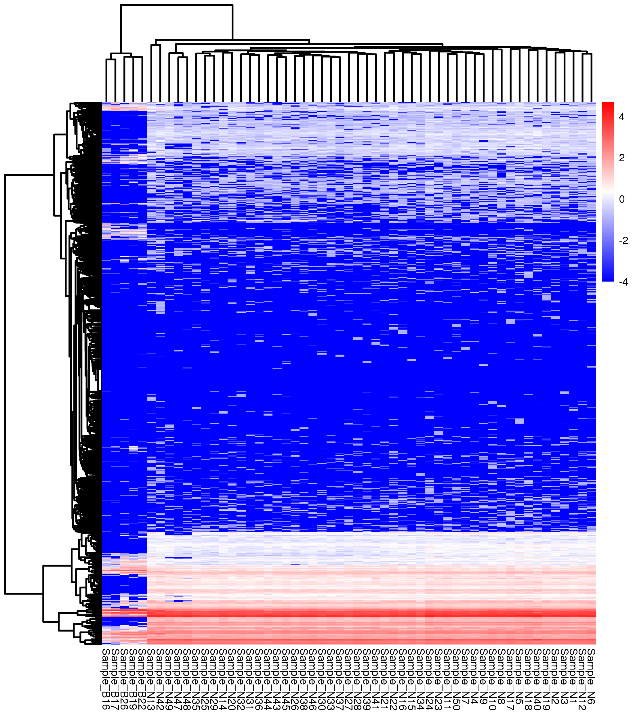

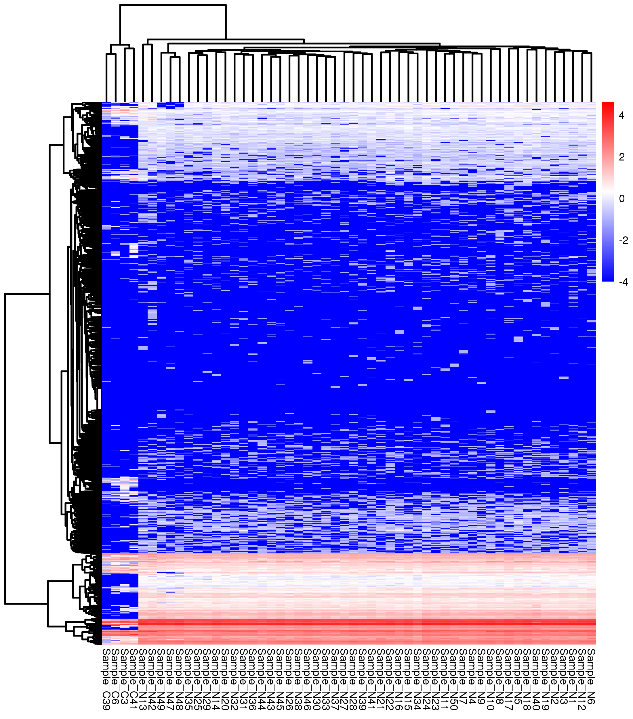


Figure. S7. Heat map of differentially expressed miRNAs in CCA or GBC patients derived exosomes

**a** Heat map of differentially expressed miRNAs in CCA patients derived exosomes.

**b** Heat map of differentially expressed miRNAs in GBC patients derived exosomes.

Up-regulated miRNAs in Cholangiocarcinoma

Up-regulated miRNAs in Gallbladder carcinoma

**a**

**b**

Figure. S8. Ten upregulated miRNAs in CCA and GBC patients derived exosomes

**a** Ten upregulated miRNAs in CCA patients derived exosomes.

**b** Ten upregulated miRNAs in GBC patients derived exosomes.

Down-regulated miRNAs in Cholangiocarcinoma

Down-regulated miRNAs in Gallbladder carcinoma

**a**

**b**

Figure. S9. Ten downregulated miRNAs in CCA and GBC patients derived exosomes

**a** Ten downregulated miRNAs in CCA patients derived exosomes.

**b** Ten downregulated miRNAs in GBC patients derived exosomes.

**a**

**c**

**d**

**b**

Figure. S10. Expression validation of 5 upregulated miRNAs in GBC patients derived exosomes

**a** Expression level of miR-151a-5p, miR-182-5p, miR-191-5p, miR-192-5p and miR-21778 in the plasma from 40 health individuals and 24 GBC patients.

**b** Receiver operating characteristic (ROC) curve analysis for GBC diagnosis. Area under the curve (AUC) estimation for miR-151a-5p, miR-182-5p, miR-191-5p, miR-192-5p and miR-21778 in GBC patients and health individuals.

**c** Expression level of miR-151a-5p, miR-182-5p, miR-191-5p, miR-192-5p and miR-21778 in the plasma from 24 GBC patients according to their tumor stage.

**d** Changes of plasma levels of miR-151a-5p, miR-182-5p, miR-191-5p, miR-192-5p and miR-21778 in 24 GBC patients before (pre-Op) and 7 days after (7 day post-Op) surgical removal of the tumor.

**a**

**b**


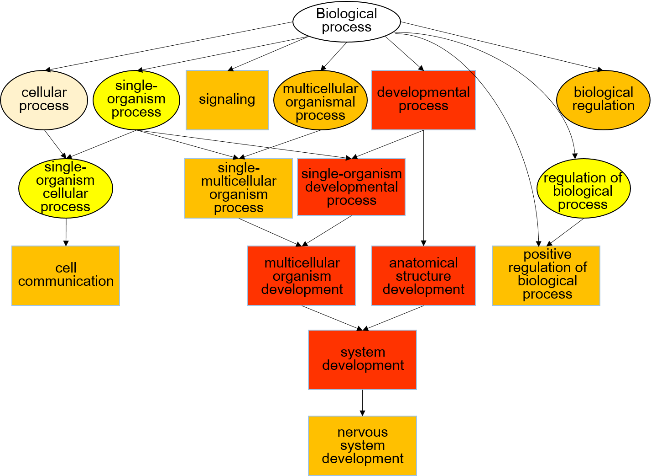

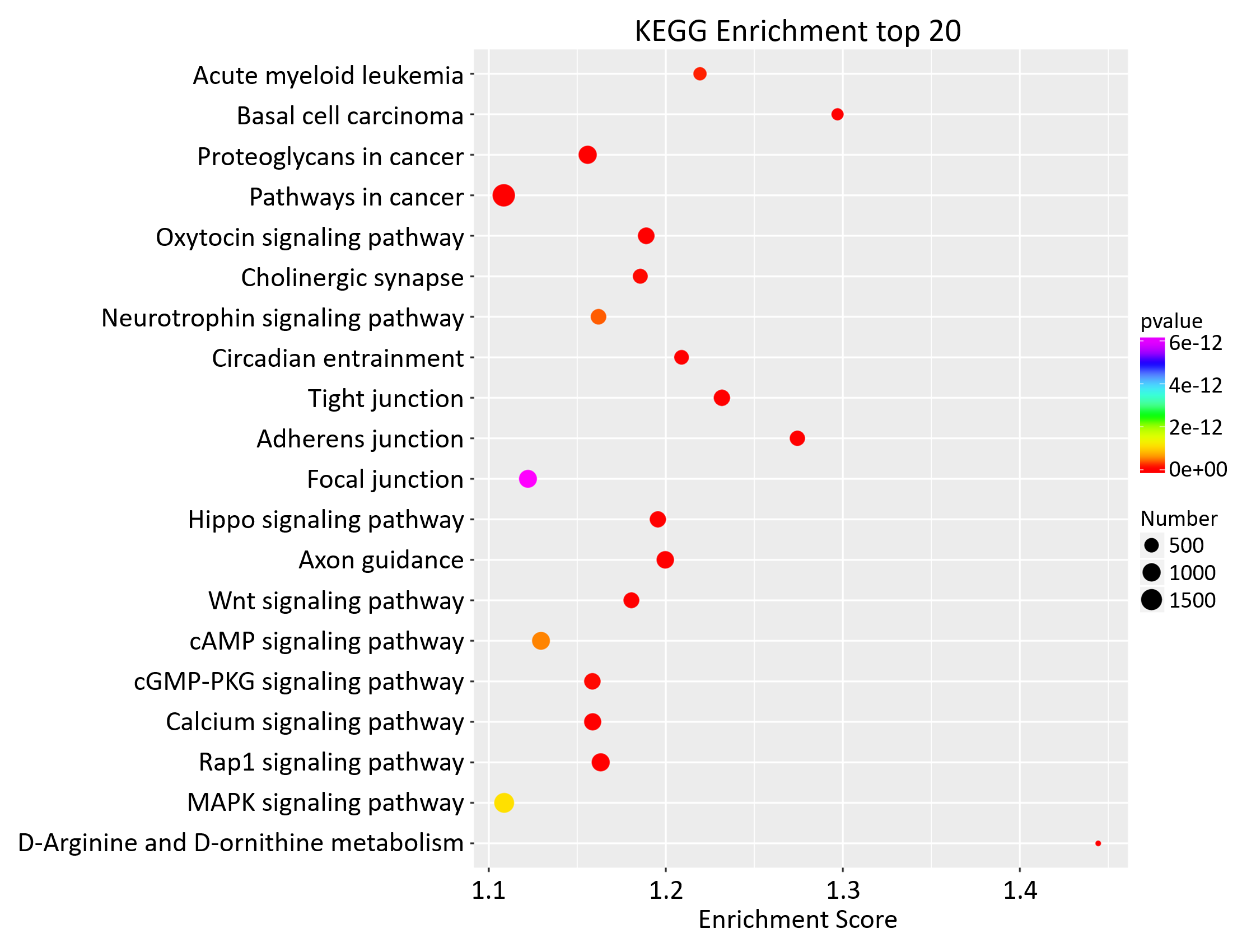


**c**

**d**


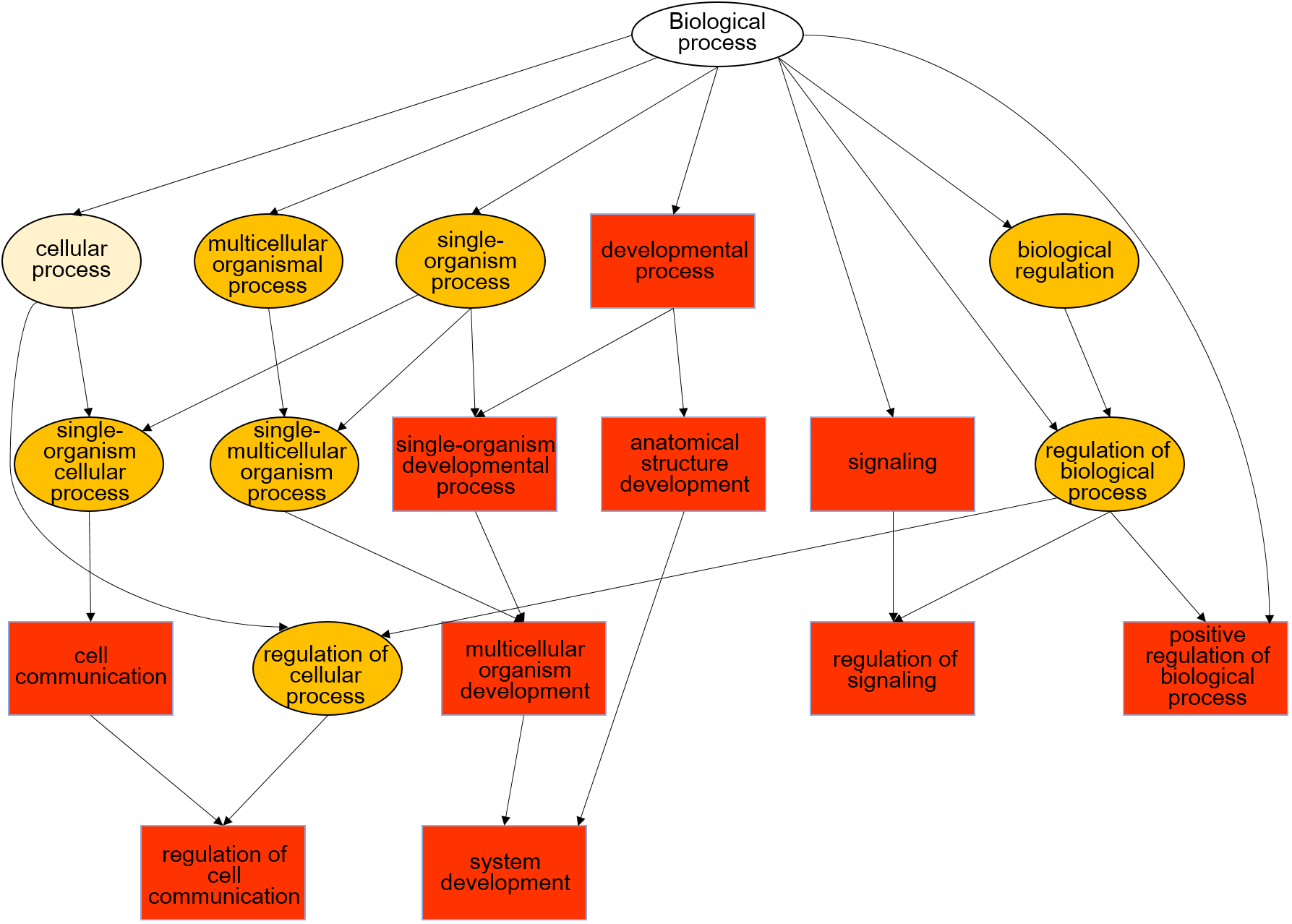

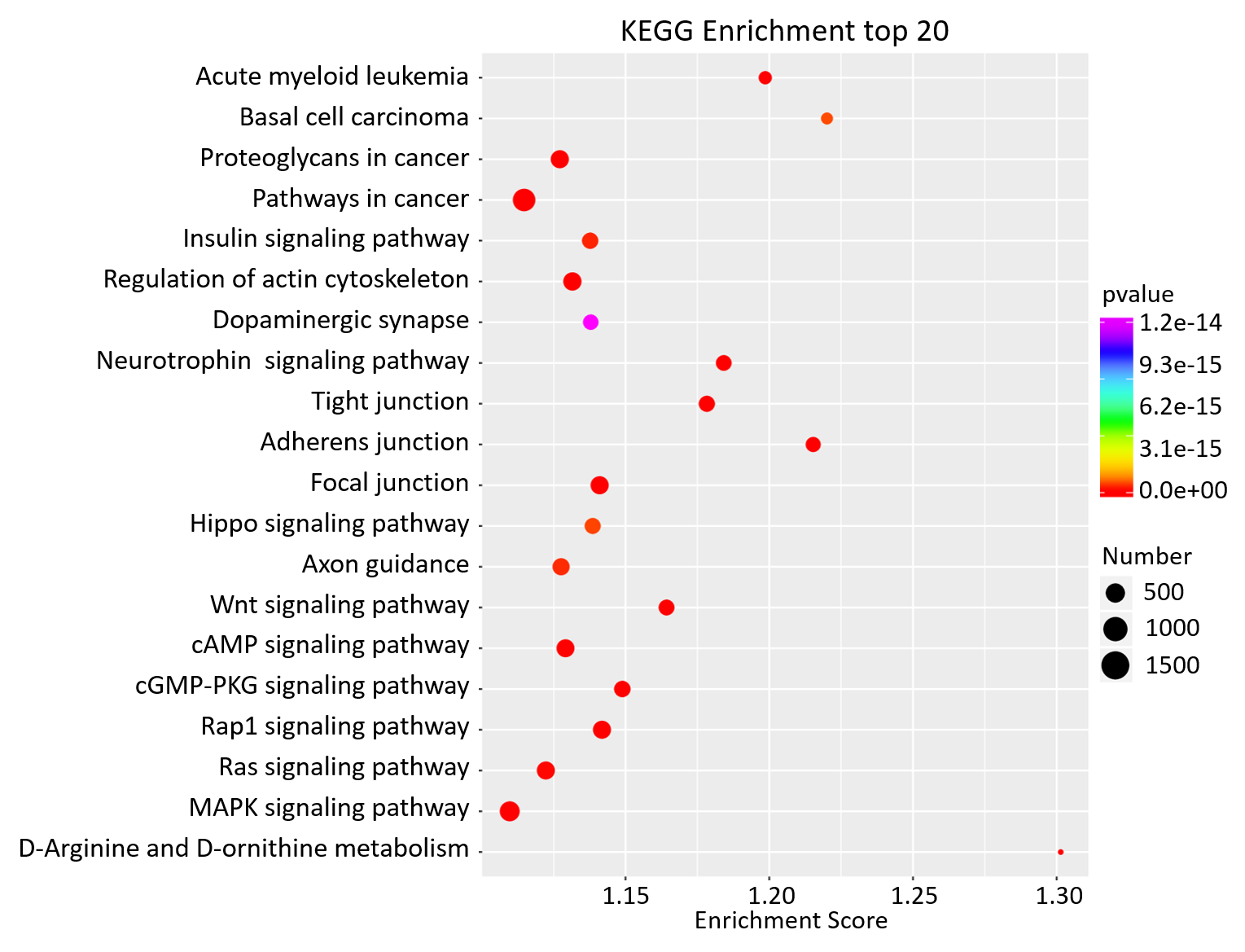


Figure. S11. KEGG and GO analysis of targets of altered miRNAs in CCA and GBC patients derived exosomes

**a** KEGG analysis of targets of altered miRNAs in CCA patients derived exosomes.

**b** GO analysis of targets of altered miRNAs in CCA patients derived exosomes.

**c** KEGG analysis of targets of altered miRNAs in GBC patients derived exosomes.

**d** GO analysis of targets of altered miRNAs in GBC patients derived exosomes.

**a**

**b**

**c**

**d**

Figure. S12. KEGG pathway analysis of targets of miR-96-5p, miR-151a-5p, miR-191-5p and miR-4732-3p

a-d KEGG pathway analysis of targets of miR-96-5p (a), miR-151a-5p (b), miR-191-5p (c) and miR-4732-3p (d). All target genes are predicted from www.targetscan.org.

**a**

**b**

**c**

**d**

Figure. S13. GO (biological process) analysis of targets of miR-96-5p, miR-151a-5p, miR-191-5p and miR-4732-3p

a-d GO analysis of targets of miR-96-5p (a), miR-151a-5p (b), miR-191-5p (c) and miR-4732-3p (d). All target genes are predicted from www.targetscan.org.


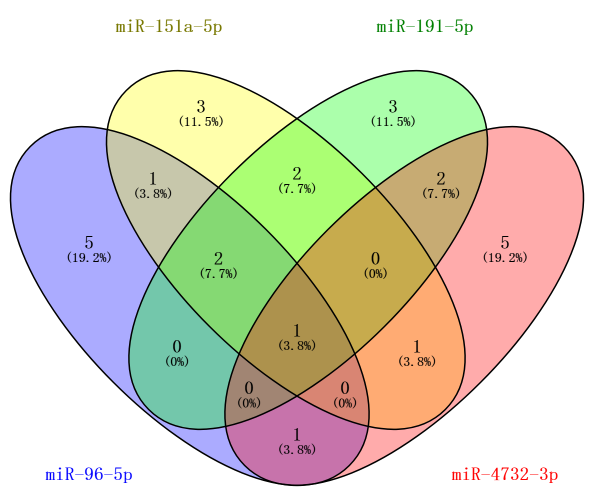

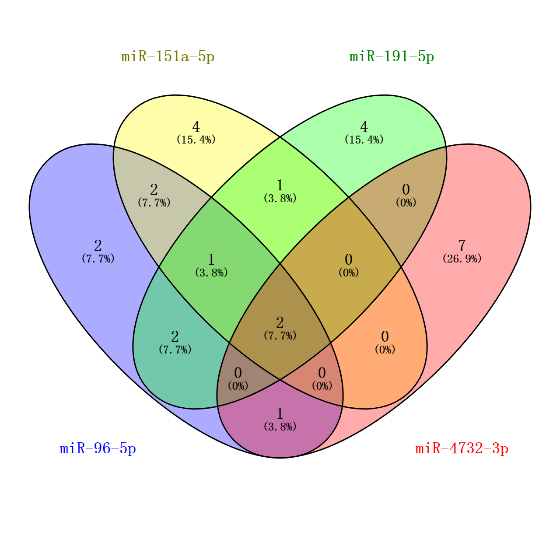


**a**

**b**

Figure. S14. Common KEGG and GO analysis of targets of miR-96-5p, miR-151a-5p, miR-191-5p and miR-4732-3p

**a** Common KEGG analysis of targets of miR-96-5p, miR-151a-5p, miR-191-5p and miR-4732-3p

**b** Common GO analysis of targets of miR-96-5p, miR-151a-5p, miR-191-5p and miR-4732-3p

Table S1. Patients information

| Sex | Age | Type | Sample number | Pathological type | Position |
| --- | --- | --- | --- | --- | --- |
| Male | 61 | CCA | B16 | Poorly differentiated adenocarcinoma | Lower bile duct |
| Male | 55 | CCA | B17 | Moderately-poorly differentiated adenocarcinoma | Hilar CCA |
| Male | 56 | CCA | B19 | Moderately differentiated adenocarcinoma | Middle bile duct |
| Male | 63 | CCA | B22 | Moderately-poorly differentiated adenocarcinoma | Middle bile duct |
| Female | 65 | CCA | B26 | Moderately differentiated adenocarcinoma | Lower bile duct |
| Male | 41 | GBC | C3 | Moderately differentiated adenocarcinoma | Gall bladder |
| Female | 56 | GBC | C6 | Moderately differentiated adenocarcinoma |  |
| Female | 52 | GBC | C39 | Moderately-poorly differentiated adenocarcinoma |  |
| Female | 64 | GBC | C41 | Moderately differentiated adenocarcinoma |  |

Reference

1 Xue, X. *et al.* Exosomal miRNA profiling before and after surgery revealed potential diagnostic and prognostic markers for lung adenocarcinoma. *Acta Biochim Biophys Sin (Shanghai)*. **52**, 281-293, (2020).

2 Yuan, T. *et al.* Plasma extracellular RNA profiles in healthy and cancer patients. *Sci Rep*. **6**, 19413, (2016).
